# Mechanisms of USP18 specificity toward ISG15 revealed by paralog sequence analysis comparison

**DOI:** 10.1016/j.jbc.2025.110288

**Published:** 2025-05-26

**Authors:** Thomas Bonacci, Derek L. Bolhuis, Nicholas G. Brown, Michael J. Emanuele

**Affiliations:** 1 Department of Pharmacology and Lineberger Comprehensive Cancer Center, The University of North Carolina at Chapel Hill, Chapel Hill, North Carolina, USA; 2 Department of Biochemistry and Biophysics and Lineberger Comprehensive Cancer Center, The University of North Carolina at Chapel Hill, Chapel Hill, North Carolina, USA

**Keywords:** USP18, ISG15, USP41, deISGylating enzymes, deubiquitinating enzymes (DUBs), alphafold

## Abstract

The ubiquitin-like protein ISG15 is activated in response to type 1 interferons, and its conjugation to proteins regulates the response to bacterial and viral infection. Its subsequent deconjugation, which is broadly achieved by the human enzyme USP18, critically controls interferon signaling and the defense against pathogens. However, the molecular determinants underlying USP18 specificity for ISG15 remain elusive. To identify such features, we took advantage of USP18's paralog USP41, which has a strikingly similar catalytic domain and yet lacks deISGylating activity. By performing a comparative sequence analysis coupled with biochemical and enzymatic assays, we identified hallmarks specific to USP18 that are critical for its enzymatic function and ISG15 recognition. Accordingly, AlphaFold-guided analysis suggests that these features mediate USP18-ISG15 interactions, underlining their importance for deISGylating activities. Thus, our results reveal important mechanistic insights into USP18-mediated ISG15 hydrolysis and could inform the development of deISGylase inhibitors relevant to infection and other interferon-related diseases.

Post-translational modifications (PTMs) by members of the ubiquitin-like family of proteins is a fundamental aspect of cell signaling involved in many biological processes (1). The founding member of this family, ubiquitin, is a small 8.5-kDa protein that gets conjugated to other proteins through a cascade of enzymes termed E1, E2, and E3, which typically results in the covalent modification of a substrate with ubiquitin onto a lysine residue (2), although ubiquitination of serine, threonine, and cysteine residues, as well as non-protein substrates, have been described (3, 4, 5). Formation of a poly-ubiquitin chain on a protein substrate often leads to its degradation by the proteasome (2). However, ubiquitination can also regulate protein-protein interactions, endocytosis, protein function, or localization (1).

Ubiquitin-like proteins (Ubls) add another layer of complexity to this signaling apparatus (6). These proteins share some sequence similarity to ubiquitin, as well as its three-dimensional structure (6). The most well-known ubiquitin-like proteins include Nedd8 (7) (neural precursor cell expressed, developmentally down-regulated 8) and the SUMO (8) (small ubiquitin-like modifier) family of proteins. However, the first Ubl that was shown to present the key feature of becoming covalently conjugated to intracellular proteins was ISG15 (interferon-stimulated gene 15), in a process called ISGylation (9, 10). ISG15 structurally resembles two ubiquitin-like molecules that each share ∼30% sequence similarity to ubiquitin and which are connected by a short linker, or hinge, region (11). While ubiquitin, Nedd8, and SUMO are all constitutively expressed in cells, ISG15 is strongly induced by type I interferons (9), such as IFN-α and IFN-β. The expression of the ISG15 conjugation machinery is also induced by interferon production, and this enzymatic cascade includes the E1 activating enzyme UBE1L/UBA7 (12, 13), the E2 conjugating enzyme Ube2L6/UbcH8 (14, 15), and HERC5 acting as the major E3 ligase (16, 17), although a couple of other E3s have been described, such as ARIH1 (18) and EFP (estrogen-responsive finger protein, also known as TRIM25 (19, 20). In contrast to ubiquitin, ISGylation is not a proteolytic signal, and ISG15 gets conjugated as monomers on target proteins (21). The main function of ISGylation is to contribute to the host response following bacterial (22) and viral infections (12). This is modulated in large part through co-translational attachment of ISG15 to viral capsid proteins, as well as host proteins, which ultimately inhibits virus assembly and/or replication (23). Other agents causing ISG15 expression include lipopolysaccharide (LPS) (24), retinoic acid (25), and certain genotoxic stressors (26), all of which illustrate the role of ISG15 in triggering a host immune response to pathogenic stimuli.

As with other Ubls, ISGylation can be reversed through the action of cysteine proteases, which include the cross-reactive deubiquitinating enzymes USP16 (27), USP21 (28), USP24 (29), and the main ISG15-specific enzyme USP18 (30). Like the rest of the ISG15 machinery, USP18 expression is triggered by type I interferons (24) and acts as a negative regulator of interferon signaling through two modes of action. First, in an enzymatic-independent mechanism, USP18 competes with Janus kinase 1 (JAK1) for interaction with the second chain of the interferon α/β receptor 2 (IFNAR2) complex, leading to downregulation of the JAK-STAT pathway (31). The importance of this scaffolding function of USP18 is illustrated by human patients harboring USP18 mutations, which give rise to interferonopathies, such as pseudo-TORCH syndrome, whose characteristic is the loss of interferon signaling (32). Second, as an ISG15-specific protease, USP18 functions as the main deISGylating enzyme *in vivo*, since mouse USP18 (mUSP18, also named Ubp43) knock-out cells display global accumulation of ISGylated proteins (24), while USP18 overexpression leads to a dramatic decrease of protein ISGylation (33).

Thus, USP18 appears to show very little substrate specificity and removes ISG15 from virtually any protein to negatively regulate the response to type I interferons (30). In addition to cleaving the covalent bond between ISG15 and a target protein, USP18 can also interact non-covalently with ISG15, which leads to accumulation of USP18 protein levels (34). However, the basis for these modes of regulation is still unknown, and the molecular determinants responsible for the specificity of USP18 toward ISG15 are not well understood. While a structural study offered insights into how the catalytic domain of mUSP18/Ubp43 engages mISG15 by identifying a motif called ISG15-binding box 1 (IBB1) (35), mUSP18/Ubp43 exhibits key functional differences compared to its human homolog. Notably, human USP18, but not mUSP18/Ubp43, is stabilized by ISG15 expression in cell-based assays and binds tightly to ISG15 non-covalently when precipitated from cells (34), together suggesting that additional mechanisms might contribute to their Ubl selectivity.

To better understand the molecular basis for the specificity of human USP18 towards ISG15, we used a mass-spectrometry-based approach and identified USP41 as a USP18 binding protein. Interestingly, USP41 is a recently evolved paralog of USP18, present in humans, but not, for example, in mice, which possess the single USP18 ortholog, mUSP18/Ubp43. Human USP18 and USP41 have very high sequence identity, particularly in their catalytic domains, which share 97% sequence similarity. Yet, remarkably, we found that USP41 does not act as an ISG15 protease. A comparative analysis, which combined molecular biology, biochemistry, and *in vitro* enzymatic assays, allowed us to illuminate previously unknown mechanisms of USP18 specificity toward ISG15. We propose that these newly identified molecular determinants of USP18 deISGylation activity are likely to represent key contact points for binding to ISG15.

## Results

### Identification of the uncharacterized DUB USP41 as a USP18-interacting protein

**Figure 1:**
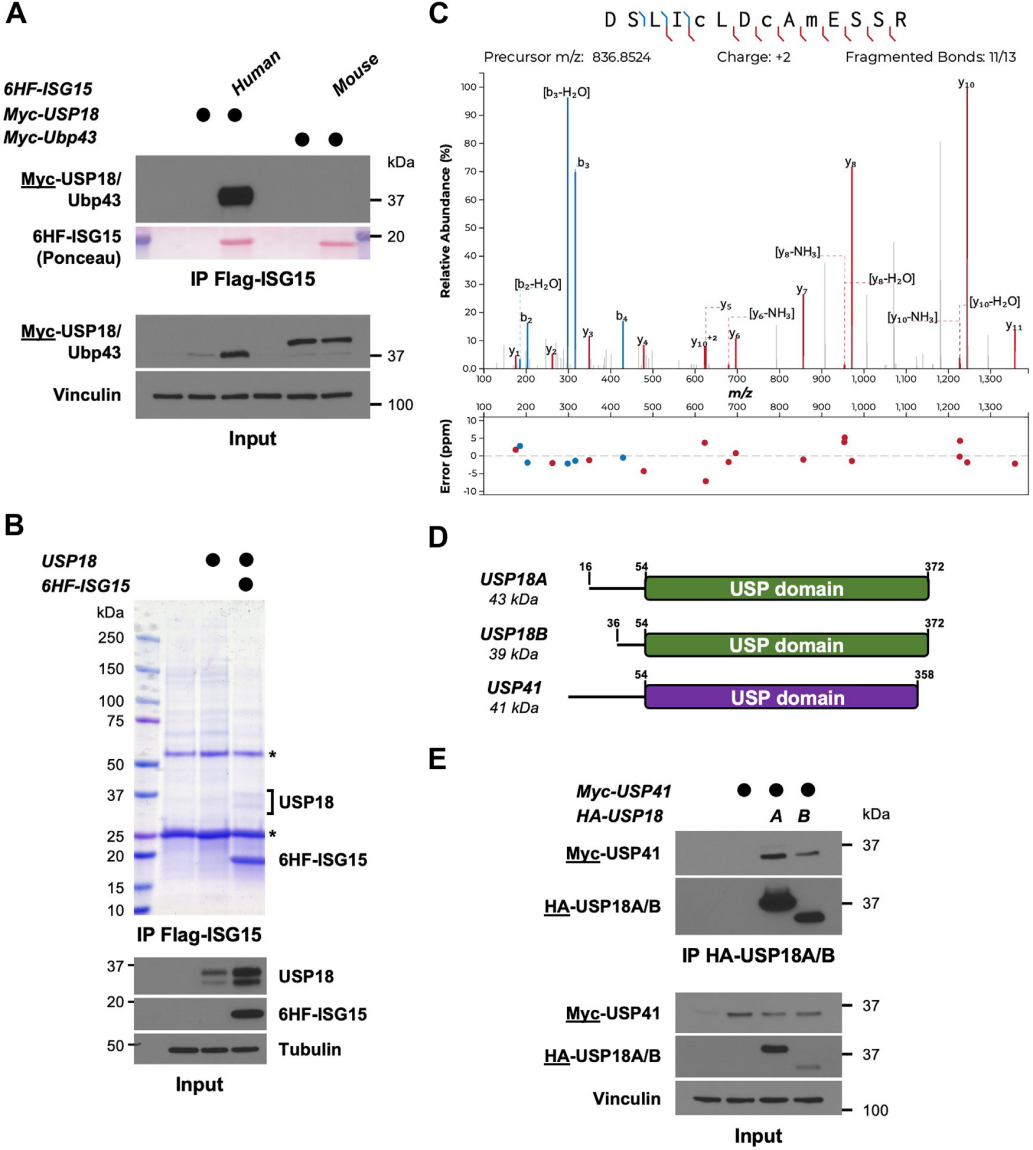
**Identification of the uncharacterized DUB USP41 as a USP18 interacting protein**. *A*, Myc-USP18, or its mouse ortholog Myc-Ubp43, were ectopically expressed in HEK-293T cells alone or with human or mouse ISG15, respectively. After 24 h, precleared lysates of transfected cells were subjected to immunoprecipitation on anti-FLAG beads. IP and input samples were separated by SDS-PAGE and analyzed by western blotting. Immunoblotted antigen is underlined to the *left* of blots. Representative of >3 independent, biological replicates. *B*, untagged USP18 was ectopically expressed in HEK-293T cells, either alone or with a 6His-FLAG-tagged ISG15 (6HF-ISG15). After 48 h, precleared lysates were used to immunoprecipitate ISG15 on anti-FLAG beads. Immunoprecipitates were separated by SDS-PAGE and the gel was stained using Colloidal Coomassie Blue staining, while inputs were analyzed by western blotting. The two USP18 isoforms that co-precipitated with ISG15 were excised, digested with trypsin, and sent for mass-spectrometry analysis. *Asterisks* indicate heavy and light chains of the FLAG antibody used for IP. Representative of >3 independent, biological replicates. *C*, MS/MS spectrum of the doubly charged ion (m/z 836.8524) corresponding to USP41 tryptic peptide DSLICLDCAMESSR. Carbamidomethylation is present on C5 and C8 corresponding to C178 and C181 in USP41. Oxidation is present on M10 corresponding to M183 in UPS41. *D*, cartoon showing the organization of USP18A, USP18B and USP41. *E*, Myc-USP41 and HA-USP18 (both isoforms) were ectopically expressed in HEK-293T cells. After 24 h, precleared lysates of transfected cells were used to immunoprecipitate USP18A and B on anti-HA beads. IP and input samples were separated by SDS-PAGE and analyzed by western blotting. Immunoblotted antigen is underlined to the *left* of blots. Representative of >3 independent, biological replicates.

The non-covalent interaction between USP18 and ISG15 is well-documented (34, 36, 37, 38), and interestingly, by using cell-based pulldown assays, the human proteins bind each other much more tightly compared to their murine counterparts, since mUSP18/Ubp43 is not co-precipitated by murine ISG15 from mammalian cell extracts (Fig. 1*A*). Likewise, the stabilization of USP18 by ISG15 is also unique to humans, as previously reported (34) (Fig. 1*A*, Input panel). We hypothesized that post-translational modifications could mediate USP18 binding to ISG15. To identify PTMs on human USP18 when it is in complex with human ISG15, we co-expressed in HEK-293T cells a 6His-FLAG-tagged version of ISG15 (6HF-ISG15), and an untagged version of USP18, which leads to expression of both USP18 isoforms (39) (Fig. 1*B*, Input panel).

Anti-FLAG immunoprecipitates were separated by SDS-PAGE and stained by Colloidal Coomassie Blue, confirming that the two isoforms of USP18 co-precipitated with ISG15 (Fig. 1*B*). The Coomassie-stained bands of both USP18 isoforms were analyzed by mass-spectrometry, with the corresponding areas in the USP18-only condition used as negative controls. Contrary to our hypothesis, USP18 is not extensively modified while in complex with ISG15, as we only found Lys173 to be modified with a di-Gly (GG) peptide (Table S1). However, our analysis identified another member of the USP family, USP41, in the ISG15 immunoprecipitates and not in the negative control (Figs. 1*C*, S1*A*, and Tables S1 and S2, orange lines). USP41 shares significant sequence identity to USP18 and has many identical tryptic peptides, but importantly, we identified USP41-specific peptides by mass spectrometry (Fig. 1*C* and S1*A*). USP41 is a protein with a similar domain organization and molecular weight as USP18 (Fig. 1*D*). Interestingly, the protein-protein interaction databases BioPlex and BioGRID reported USP41 as a USP18-interacting protein, consistent with its detection in our mass spectrometry experiment.

USP18 exists as two isoforms that we refer to here as USP18A and USP18B, with starting sequences at positions 16 and 36, respectively, reflecting the correct expression of USP18 isoforms (Fig. S1*B* and Supplementary Document 1 for sequences) since the first 15 amino acids on the predicted USP18 N-terminus are not, in fact, part of the final gene products (39). To test whether USP41 interacts with USP18 isoforms, we expressed a Myc-tagged version of USP41, alone or in combination with HA-tagged versions of USP18A or USP18B in HEK293T cells. Immunoprecipitation of both USP18 isoforms demonstrated their interaction with USP41 (Fig. 1*E*), which was also observed by precipitating Myc-USP41 (Fig. S1*C*). From now on, we will simply use USP18 to refer to USP18A throughout the manuscript, unless noted otherwise. We next generated truncations of USP18 and USP41 lacking their respective N-termini, leaving only their USP catalytic domain (USP18 CD and USP41 CD, respectively). These truncated versions retained the ability to interact, revealing that USP41 and USP18 bind through their respective USP domains (Fig. S1, *D* and *E*). Together, our results identify USP41 as a new USP18 interacting protein.

### USP41 is a paralog of USP18, which does not react with ISG15

**Figure 2:**
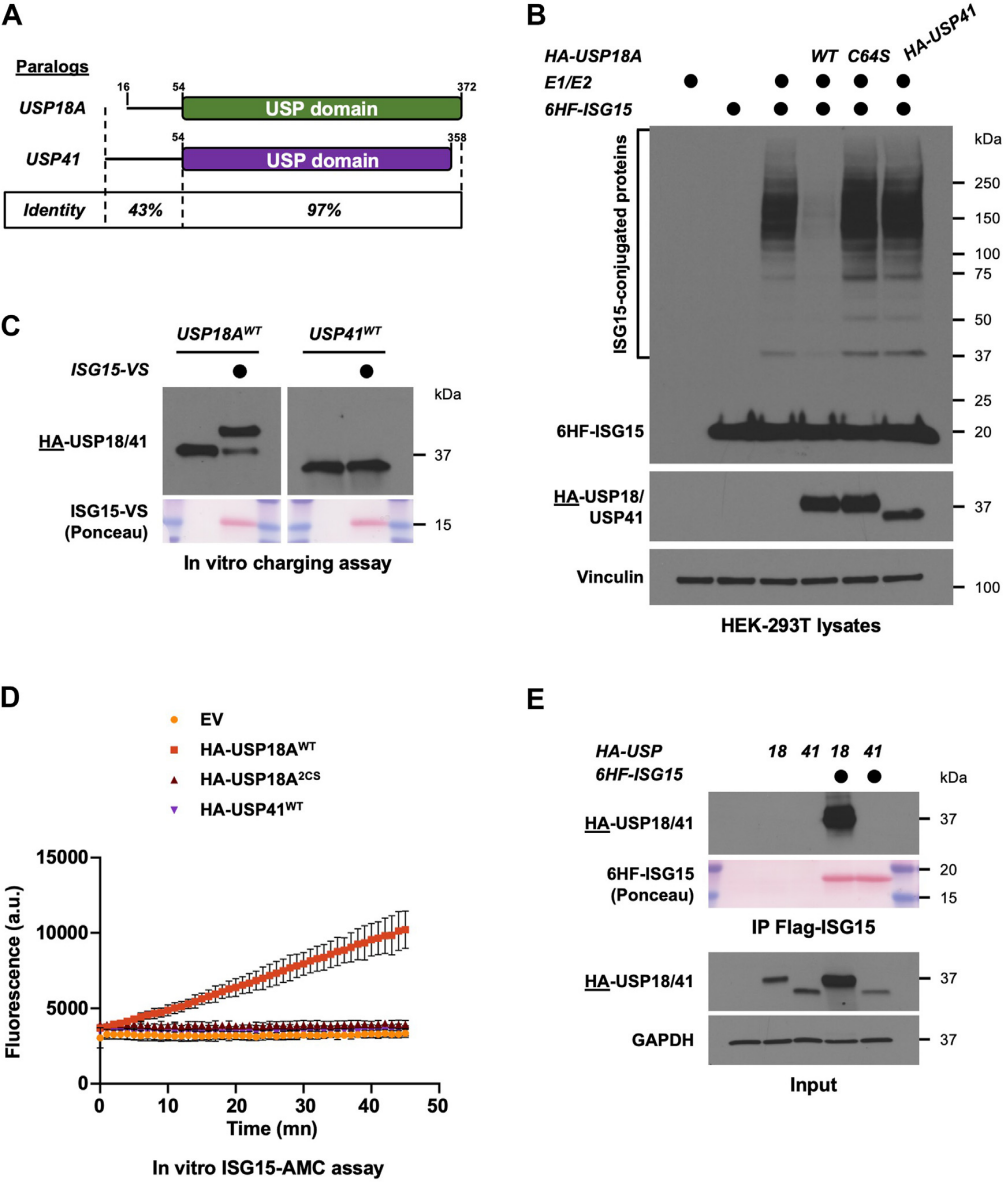
**USP41 is a paralog of US18 which does not react with ISG15.***A*, cartoon showing the sequence identity of USP18 and USP41. *B*, protein ISGylation in HEK-293T cells was reconstituted by ectopically expressing V5-UBA7 (E1), V5-UbcH8 (E2) and 6His-FLAG-ISG15 (6HF-ISG15). Where indicated, HA-USP18 WT, a catalytically inactive version (C64S), or HA-USP41, were co-transfected. After 24 h, lysates of transfected cells were prepared then analyzed by SDS-PAGE and Western blot. Immunoblotted antigen is underlined to the *left* of blots. Representative of >3 independent, biological replicates. *C*, HA-USP18 and HA-USP41 were ectopically expressed in HEK-293T cells and after 24 h, precleared lysates of transfected cells were used to immunoprecipitate USP18 or USP41 on anti-HA beads. Immunoprecipitates were mixed with reaction buffer containing an ISG15 activity-based probe (ISG15-VS) or not, and reaction products were analyzed by SDS-PAGE and Western blot. Representative of >3 independent, biological replicates. *D*, the indicated constructs were ectopically expressed in HEK-293T cells and after 24 h, precleared lysates of transfected cells were used to immunoprecipitate USP18 WT, USP18 2CS, or USP41 WT on anti-HA beads. Immunoprecipitates were mixed with reaction buffer containing the fluorogenic substrate ISG15-AMC, and fluorescence increase was monitored using a plate reader at the appropriate excitation and emission wavelengths. Representative of >3 independent, biological replicates. *E*, HA-USP18 and HA-USP41 were ectopically expressed in HEK-293T cells, either alone or with 6His-FLAG-ISG15. After 24 h, precleared lysates of transfected cells were used to immunoprecipitate ISG15 on anti-FLAG beads. Immunoblotted antigen is underlined to the *left* of blots. Representative of >3 biological replicates.

USP41 is a largely uncharacterized DUB, however, a sequence analysis revealed that it is a close paralog of USP18, as illustrated by their very high sequence identity (Fig. 2*A* and Supplementary Document 1). The catalytic domains of USP41 and USP18 share a remarkable 97% identity which led us to hypothesize that USP41 could be a second ISG15 protease and therefore, we tested whether USP41 could remove ISG15 from endogenous proteins.

Co-expression of 6HF-ISG15, together with its E1 (V5-UBA7) and E2 (V5-UbcH8) leads to the conjugation of ISG15 onto endogenous proteins, and this is not observed if the E1 is omitted, if the E2 is catalytically inactive (C86A), or if a non-conjugatable mutant of ISG15 (ΔGG) is used (Fig. S2*A*). Using this system, co-expression of USP18 led to the almost complete deconjugation of ISG15 from endogenous proteins, and this is dependent on its catalytic cysteine (Fig. 2*B*, compare USP18 WT and C64S lanes). Strikingly, co-expression of USP41 did not affect ISGylation in HEK-293T cells (Fig. 2*B*). However, mUSP18/Ubp43 was able to deconjugate human ISG15 from human proteins as efficiently as human USP18 (Fig. S2*B*). This is notable because mUSP18/Ubp43 has comparatively less sequence similarity to human USP18 relative to USP41 (Fig. S2*C*).

Next, we aimed to assess the potential activity of USP41 towards ISG15. While ideally achieved using a recombinant expression system, USP18 is difficult to express *in vitro* (40). So far, only the catalytic domain of mUSP18/Ubp43, lacking its N-terminus sequence, has been successfully made using a baculoviral expression system (35), further illustrating the challenge of generating recombinant versions of human USP18. We devised a strategy to express and purify human USP18 and USP41 from HEK293T cells using immunoprecipitation, and to use these immunoprecipitates for enzymatic assays (see Experimental procedures). This approach circumvents the limitations of recombinant expression systems and allows for the small-scale purification of many different versions of mUbp43, USP18, and USP41.

We used this approach to perform two complementary *in vitro* assays to monitor enzymatic activity. First, we used an ISG15 activity-based probe (ABP), ISG15-vinyl sulfone (ISG15-VS), whose C-terminal vinyl sulfone warhead forms a covalent bond with the active site cysteine of a protease, allowing us to monitor enzymatic activity. HA-tagged versions of USP18 or USP41 were isolated from HEK-293T cells and then mixed with ISG15-VS or buffer as a control. USP18 readily reacted with the ABP, as illustrated by its shift in size on SDS-PAGE (Fig. 2*C*). In contrast, USP41 did not react with the ISG15-VS ABP. Second, we used the fluorogenic substrate ISG15-AMC which generates fluorescence following AMC cleavage by a protease. Similar immunoprecipitates of HA-tagged WT USP18, catalytically inactive USP18, or WT USP41 were mixed with the ISG15-AMC. We observed an increase in fluorescence when the fluorogenic substrate was mixed with WT USP18, indicative of protease-mediated cleavage of AMC from ISG15. However, there was no detectable activity in the presence of either USP41 or a catalytically inactive version of USP18 (Figs. 2*D* and S2*D* for control of expression).

Finally, we examined whether USP41 could bind ISG15, since USP18 non-covalently interacts with ISG15. HA-tagged versions of USP18 and USP41 were expressed alone or in combination with 6HF-ISG15, and following FLAG-IP, we could readily detect USP18 interacting non-covalently with ISG15, whereas USP41 did not (Fig. 2*E*). Moreover, ISG15 expression led to a stabilization and increased abundance of USP18 (Fig. 2*E*, Input panel), but it did not affect USP41. Thus, USP18 binds, is stabilized by, and can deconjugate ISG15 based on multiple enzymatic assays. In contrast, USP41 can neither bind, be stabilized by, nor deconjugate ISG15. This data strongly suggests that, despite being a paralog of USP18, USP41 is not an ISG15 protease, despite having catalytic domains that bear 97% sequence identity to one another.

### The C-terminus of USP18 is necessary for its enzymatic activity towards ISG15

**Figure 3:**
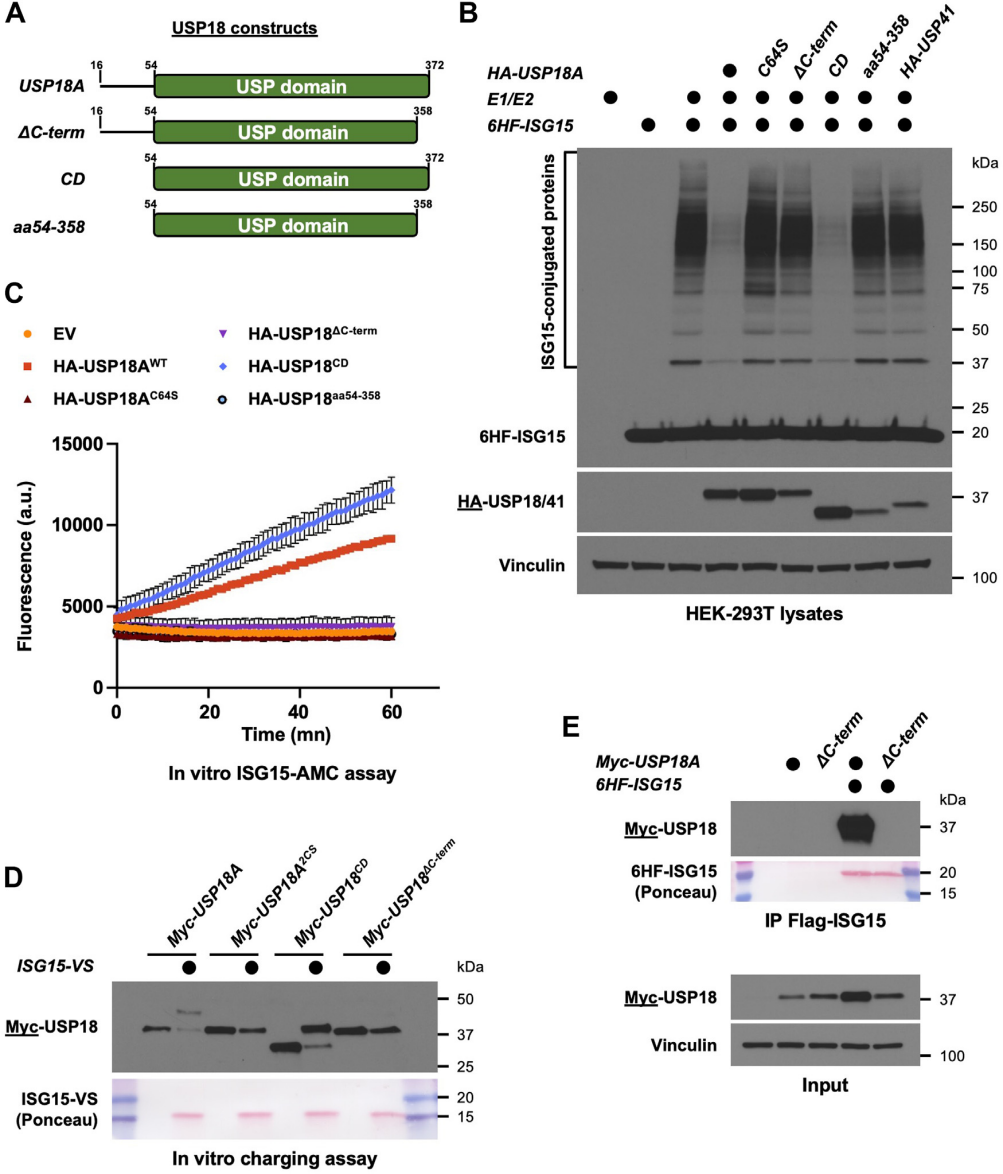
**The C-terminus of USP18 is necessary for its function**. *A*, cartoon showing the different truncation constructs of USP18 that were generated. The ΔC-term lacks the last 14 amino acids of the USP domain, and the CD leaves only the catalytic domain. The construct aa54 to 358 represents USP18 catalytic domain without the last 14 amino acids. *B*, protein ISGylation in HEK-293T cells was reconstituted by transfection of the ISG15 machinery (E1/E2/ISG15) and where indicated, HA-tagged USP18 constructs were co-transfected. After 24 h, lysates of transfected cells were prepared then analyzed by SDS-PAGE and Western blot. Immunoblotted antigen is underlined to the *left* of blots. Representative of >3 independent, biological replicates. *C*, the indicated constructs were ectopically expressed in HEK-293T cells and after 48 h, precleared lysates of transfected cells were used to immunoprecipitate HA-tagged USP18 variants on anti-HA beads. Immunoprecipitates were mixed with reaction buffer containing the fluorogenic substrate ISG15-AMC, and fluorescence increase was monitored as previously described. Representative of >3 independent, biological replicates. *D*, the indicated Myc-tagged USP18 constructs were ectopically expressed in HEK-293T cells and after 48 h, precleared lysates of transfected cells were used to immunoprecipitate USP18 on anti-Myc beads. Immunoprecipitates were mixed with reaction buffer containing ISG15-VS or not, and reaction products were analyzed by SDS-PAGE and Western blot. Representative of >3 independent, biological replicates. *E*, HA-USP18 FL or HA-USP18 ΔC-term were ectopically expressed in HEK-293T cells, either alone or with 6HF-ISG15. After 24 h, precleared lysates of transfected cells were used to immunoprecipitate ISG15 on anti-FLAG beads. Immunoblotted antigen is underlined to the *left* of blots. Representative of >3 independent, biological replicates.

Since USP41 is likely to be a gene duplication of USP18, its inability to deconjugate ISG15 suggests that evolutionary differences between their protein sequences confer the key functional determinants necessary for deISGylating activity. Given their remarkable similarity, this provided a unique opportunity for discovering molecular features that allow USP18 to perform deISGylation. When aligning their protein sequences, the most striking difference is that the USP41 catalytic domain is shorter than USP18's by 14 amino acids on its C-terminus (Fig. 2*A*). We therefore examined the impact of deleting these residues from either USP18 or its catalytic domain. We generated deletion constructs of USP18 (Fig. 3*A*) and co-expressed each of them along with the ISG15 machinery in HEK-293T cells. As previously shown, USP18 can efficiently remove ISG15 from endogenous proteins, and this is dependent on its catalytic cysteine (Fig. 3*B*, compare USP18 to C64S). Deletion of the last 14 amino acids of USP18 completely abrogated its enzymatic activity (Fig. 3*B*, compare USP18 to ΔC-term). Similarly, while the catalytic domain of USP18 retains the ability to deconjugate ISG15 *in vivo*, deleting the last 14 amino acids from the catalytic domain blocked its activity (Fig. 3*B*, compare CD to aa54–358). As a negative control, USP41 was also included and had no effect on ISG15-conjugated proteins. We also co-expressed FL, CD, or ΔC-term versions of mUSP18/Ubp43 along with the murine ISG15 machinery and observed that deleting the last 14 amino acids of mUSP18/Ubp43 completely abrogated enzymatic activity to comparable levels as the catalytically inactive mutant C61S (Fig. S3*A*). Thus, the C-terminal 14 amino acids of USP18 are crucial for its ability to deconjugate ISG15 from substrates in cells.

To test the importance of the USP18 C-terminus for its enzymatic activity *in vitro*, we immunopurified our USP18 deletion constructs from HEK-293T cells and performed *in vitro* assays using the ISG15-AMC fluorogenic activity reporter and the ISG15-VS ABP. Consistently, deleting the last 14 amino acids from either USP18 or its catalytic domain (ΔC-term or aa54–358, respectively) blocked activity toward ISG15-AMC (Figs. 3*C* and S3*D* for control of expression). Moreover, USP18 ΔC-term did not react with ISG15-VS, similar to the C64S/C65S (2CS) catalytically inactive mutant (Fig. 3*D*). Identical results were obtained for mUSP18/Ubp43 (Fig. S3*B*). Finally, we examined the non-covalent binding between USP18 and ISG15 and found that USP18 ΔC-term could not bind to ISG15 by co-IP (Fig. 3*E* and S3*C*) and was not stabilized by ISG15 co-expression (Fig. 3*E*, Input panel).

The previously solved structure of mUSP18/Ubp43 in complex with mISG15 indicates that the C-terminus is inserted into the catalytic domain, between several beta sheets (35). Therefore, we wondered if deleting the USP18 C-terminus led to misfolding and intracellular aggregation, potentially explaining the loss of enzymatic activity. Cell lysates of HEK-293T expressing either USP18 or its ΔC-term mutant were analyzed using size exclusion chromatography (SEC). The majority of USP18 was found across fractions #19 to 21 by SEC, and importantly, the ΔC-term mutant showed a similar pattern (Fig. S4). Of note, both USP18 and the ΔC-term mutant were also found in additional fractions as part of larger complexes, which might be explained by interactions with ribosomes and/or chaperones. Nevertheless, their overall similar profiles by SEC mitigate the concern that deleting the C-terminus of USP18 leads to its gross misfolding and aggregation, further supporting the importance of this sequence in mediating the deISGylation function of USP18.

### Identification of the TAIL motif as a molecular determinant of ISG15 binding

**Figure 4:**
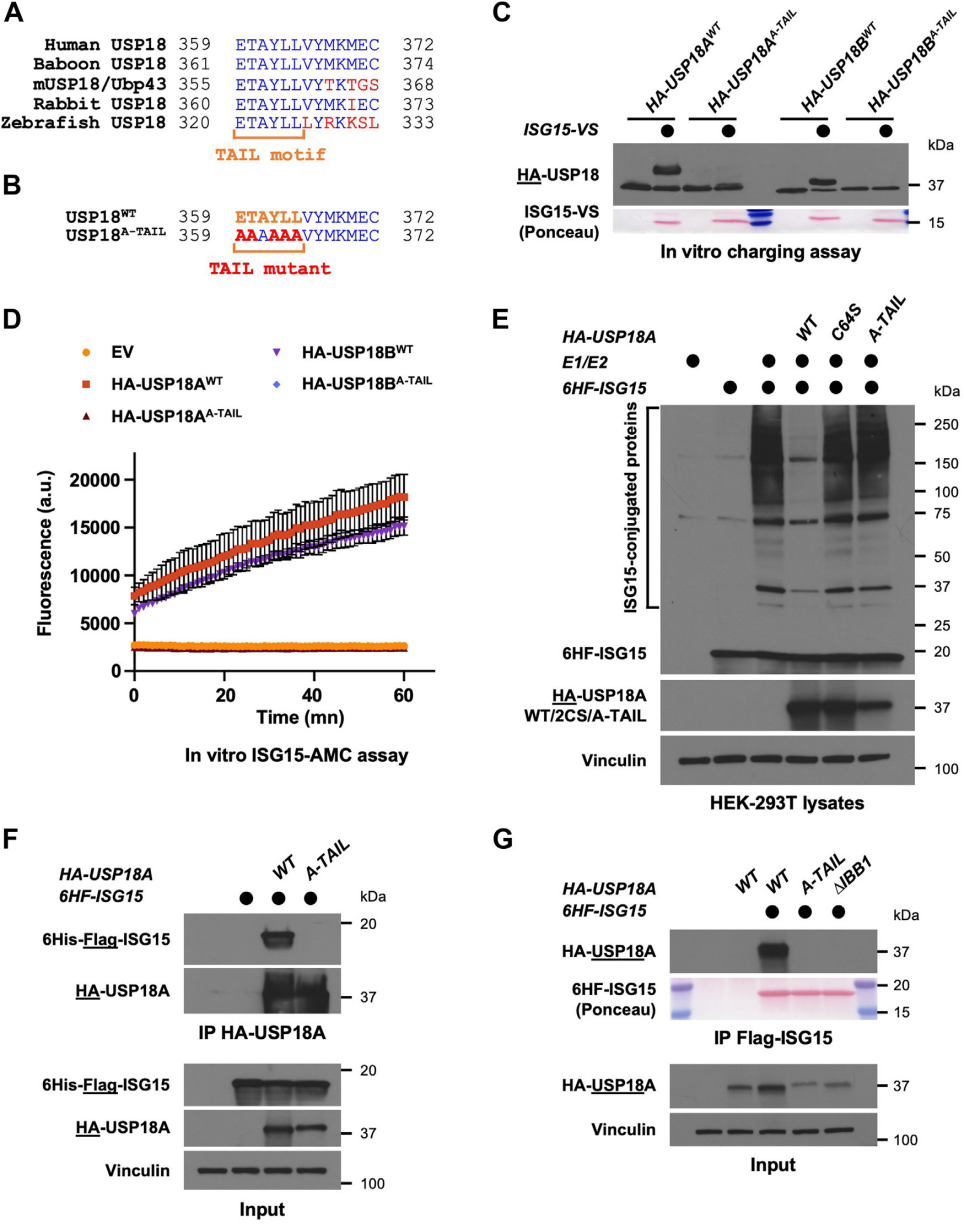
**Identification of the TAIL motif as a molecular determinant of ISG15 binding**. *A*, Sequence alignment of the C-termini of human USP18, baboon USP18, mUSP18/Ubp43, rabbit USP18 and zebrafish USP18. *Red* indicates residues that are divergent, while the TAIL motif is indicated in *orange*. *B*, sequence of USP18 A-TAIL mutant that was generated compared to its WT counterpart. *C*, HA-tagged USP18 WT or A-TAIL were ectopically expressed in HEK-293T cells then immunoprecipitated on anti-HA beads. Immunoprecipitates were mixed with reaction buffer containing ISG15-VS or not, and reaction products were analyzed by SDS-PAGE and Western blot. Representative of >3 independent, biological replicates. *D*, the indicated constructs were ectopically expressed and purified from HEK-293T cells as in (*C*). Immunoprecipitates were mixed with reaction buffer containing the fluorogenic substrate ISG15-AMC, and fluorescence increase was monitored as previously described. Representative of >3 independent, biological replicates. *E*, protein ISGylation in HEK-293T cells was reconstituted by transfection of the ISG15 machinery (E1/E2/ISG15) and where indicated, HA-tagged USP18 constructs were co-transfected. After 24 h, lysates of transfected cells were prepared then analyzed by SDS-PAGE and Western blot. Immunoblotted antigen is underlined to the *left* of blots. Representative of >3 independent, biological replicates. *F*, 6HF-ISG15 was ectopically expressed in HEK-293T cells, either alone, with WT or A-TAIL versions of HA-USP18A. At 24 h post-transfection, precleared lysates were used to immunoprecipitate HA-USP18 on anti-HA beads. Immunoblotted antigen is underlined to the *left* of blots. Representative of >3 independent, biological replicates. *G*, WT, A-TAIL or ΔIBB1 versions of HA-USP18A were ectopically expressed in HEK-293T cells in combination with 6HF-ISG15. After 24 h, precleared lysates of transfected cells were used to immunoprecipitate ISG15 on anti-FLAG magnetic beads. Immunoblotted antigen is underlined to the *left* of the blots. Representative of two independent, biological replicates.

Sequence analysis of human, baboon, mouse, rabbit, and zebrafish USP18, highlighted the presence of six evolutionary conserved residues in the C-terminal tail, which formed the sequence ETAYLL, and we named this patch the “TAIL motif” (Fig. 4*A*). Its high degree of conservation led us to test its importance for USP18's function toward ISG15. We mutated all residues to Alanine to generate mutant versions of USP18 A and B that we named USP18^A-TAIL^ (Fig. 4*B*). This revealed that these mutants were unable to react with the ABP ISG15-VS (Fig. 4*C*). Consistently, the A-TAIL mutants displayed no detectable enzymatic activity toward ISG15-AMC compared to their WT counterparts (Figs. 4*D* and S5*A* for control of expression). Next, we monitored the impact of mutating the TAIL motif in cells expressing the ISGylation machinery. Significantly, USP18A^A-TAIL^ was unable to perform deISGylation *in vivo*, whose lack of activity was comparable to that of the catalytically inactive C64S mutant (Fig. 4*E*). To determine whether this effect was due to altering USP18 enzymatic activity or its binding to ISG15, we monitored non-covalent interaction between USP18 and ISG15. Strikingly, the A-TAIL mutation completely abrogated the USP18-ISG15 interaction based on reciprocal pulldown experiments from cells transiently expressing both proteins, irrespective of which protein was precipitated (Figs. 4*F* and S5*A*). To further validate that the TAIL motif mediates binding to ISG15, we mutated a hydrophobic region of USP18 termed IBB1 (ISG15-binding box 1) which is known to be critical for the interaction between USP18 and ISG15. By performing co-immunoprecipitation experiments, we found that mutating either the TAIL motif or IBB1 similarly abrogated the interaction between USP18 and ISG15 (Fig. 4*G*). Accordingly, mutation of either the TAIL motif or IBB1 also impaired USP18's ability to react with the ISG15-VS ABP (Fig. S5*B*). Altogether, these experiments demonstrate that the TAIL motif on USP18 C-terminus plays a critical role for its function by mediating the interaction between ISG15 and USP18 and thus its ability to deconjugate ISG15.

### Engineering USP41 into a deISGylase identifies Leu198 of USP18 as crucial for activity

**Figure 5:**
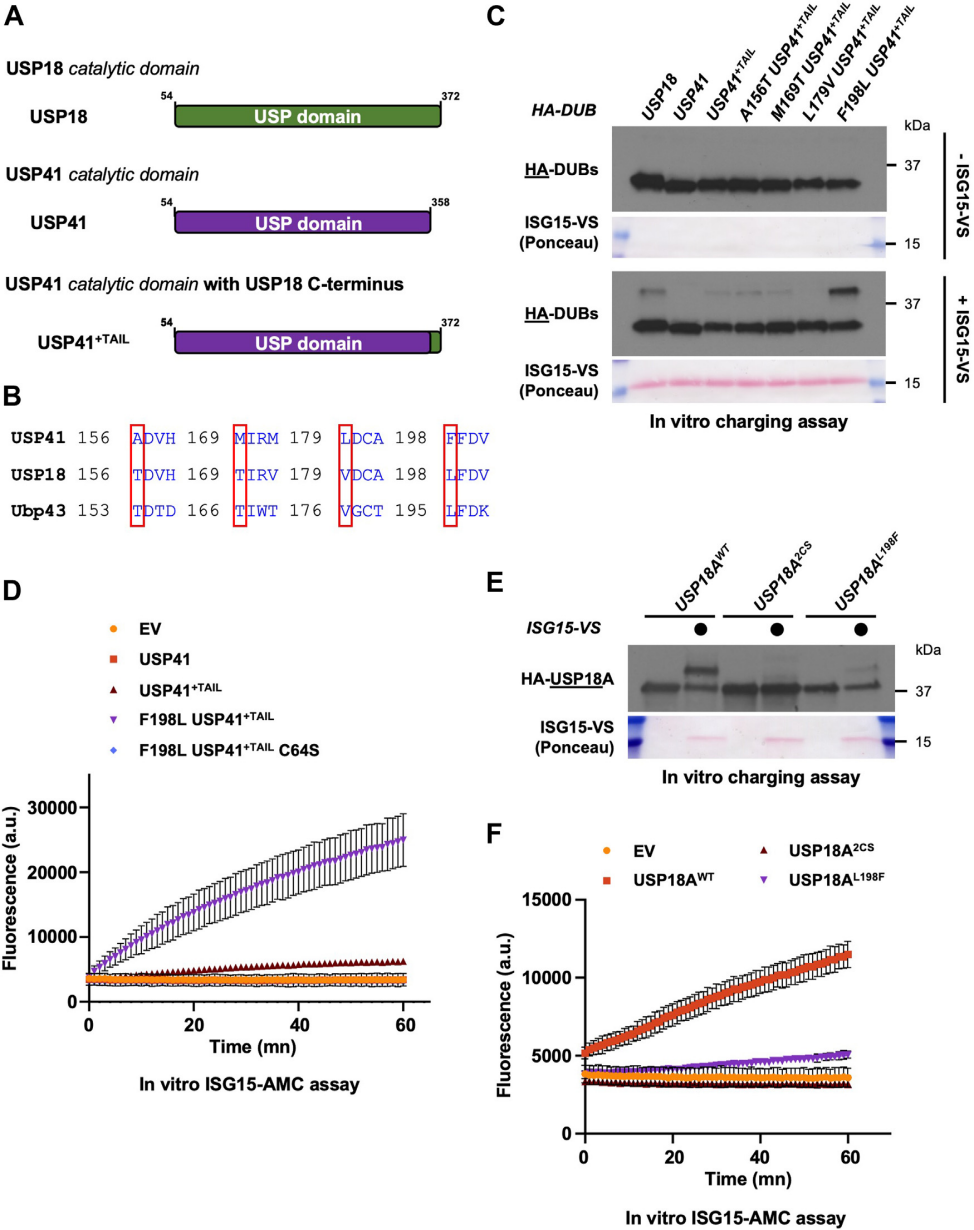
**Engineering USP41 into a deISGylase identifies Leu198 of USP18 as crucial for activity.***A*, cartoon showing the different truncation constructs of USP18 and USP41 that were generated. USP41^+TAIL^ is a chimeric construct where the last 14 amino acids of USP18 were added to the USP domain of USP41. *B*, sequence alignment of Ubp43, USP18 and USP41. *Red* boxes highlight residues that are different between USP18 and USP41 but conserved between USP18 and mUSP18/Ubp43. *C*, the indicated HA-tagged constructs were ectopically expressed in HEK-293T cells and after 24 h, precleared lysates of transfected cells were used to immunoprecipitate these HA-DUBs on anti-HA beads. Immunoprecipitates were mixed with reaction buffer containing ISG15-VS or not, and reaction products were analyzed by SDS-PAGE and Western blot. Representative of >3 independent, biological replicates. *D*, same as in (*C*), except that after immunoprecipitation, the indicated HA-tagged constructs were mixed with reaction buffer containing the fluorogenic substrate ISG15-AMC, and fluorescence increase was monitored as previously described. Representative of >3 independent, biological replicates. *E*, the indicated HA-tagged USP18A constructs were purified from HEK-293T cells on anti-HA beads 48 h after transfection. HA-USP18A immunoprecipitates were mixed with reaction buffer containing ISG15-VS or not, and reaction products were analyzed by SDS-PAGE and Western blot. Representative of >3 independent, biological replicates. *F*, the indicated Myc-tagged constructs were ectopically expressed in HEK-293T cells and after 24 h, precleared lysates of transfected cells were used to immunoprecipitate Myc-tagged USP18 variants on anti-Myc beads. Immunoprecipitates were mixed with reaction buffer containing the fluorogenic substrate ISG15-AMC, and fluorescence increase was monitored as previously described. Representative of >3 independent, biological replicates.

We next asked whether addition of the TAIL motif would be sufficient to turn USP41 into an active ISG15 protease. We used USP18 and USP41 constructs expressing their catalytic domain only (Fig. 5*A*) and a chimeric USP41 construct that is appended with the C-terminus of USP18 that we name USP41^+TAIL^ (Fig. 5*A*). Immunopurified proteins were assessed for their ability to react with the ISG15-VS ABP. Surprisingly, USP41^+TAIL^ did not react with the ISG15-VS ABP (Fig. S6*A*). USP18 and USP41^+TAIL^ share a remarkably conserved catalytic domain, that is of identical length with no missing segments. And yet, USP41^+TAIL^ lacks the ability to perform deISGylation, meaning that additional sequence features must contribute to deISGylating activity.

There are only nine amino acid differences between USP18 and USP41^+TAIL^ catalytic domains. We reasoned that amino acids that are different between USP18 and USP41^+TAIL^, but which are conserved between USP18 and mUSP18/Ubp43, would be important for carrying out deISGylation. Sequence alignment of USP18, USP41^+TAIL^, and mUSP18/Ubp43 revealed four candidate residues on USP18 and USP41^+TAIL^. In USP41, these corresponded to amino acids Ala156, Met169, Leu179, and Phe198, and in USP18 to amino acids Thr156, Thr169, Val179, and Leu198 (Fig. 5*B*). We used the USP41^+TAIL^ chimera to mutate these residues to the corresponding amino acid of USP18, and each was then assayed using the ISG15-VS ABP. Replacement of Ala156, Met169 or Leu179 on USP41 to the corresponding USP18 residues (Thr156, Thr169, or Val179, respectively) did not turn USP41^+TAIL^ into a deISGylase. However, substituting USP41 Phe198 to the corresponding Leucine of USP18 led to efficient charging of USP41^+TAIL^ with the ISG15-VS ABP (Fig. 5*C*). Importantly, the newly engineered activity of USP41 toward ISG15 was dependent on its catalytic Cys64 (Fig. S6*B*). To confirm deISGylating activity, we first tested its activity toward the ISG15-AMC fluorogenic substrate. Consistently, AMC fluorescence increased only when the chimera F198L USP41^+TAIL^ was used, and this was blocked by mutating Cys64 into serine (Figs. 5*D* and S6*C* for control of expression). Next, we co-expressed these constructs along with the ISG15 machinery in HEK-293T cells and monitored protein deISGylation. The chimera F198L USP41^+TAIL^ was also able to cleave ISG15 from proteins in a similar manner to USP18, and this was dependent on Cys64 (Fig. S6*D*). Thus, the Phenylalanine at position 198 blocks USP41^+TAIL^ activity towards ISG15.

These observations suggested a potentially key role for Leucine 198 in mediating USP18's deISGylating activity. Therefore, we generated an L198F substitution and assessed the ability of this USP18 mutant to be charged with the ISG15-VS ABP or process the ISG15-AMC reporter. Remarkably, mutating Leu198 to Phenylalanine reduced USP18's ability to react with the ISG15-VS ABP (Fig. 5*E*, compare WT to L198F). Consistently, the activity of USP18 L198F was severely impaired based on the ISG15-AMC fluorogenic reporter (Figs. 5*F* and S8*E*, compare WT to L198F). We also monitored the ability of this mutant to interact with ISG15 by performing co-immunoprecipitation experiments. Remarkably, the L198F mutant co-precipitated with ISG15 to the same extent as WT USP18 (Fig. S6*E*), further illustrating the importance of Leu198 for USP18 enzymatic activity rather than ISG15 binding. To establish the role of Leu198 in mediating USP18 activity, we also mutated this residue to phenylalanine on mUSP18/Ubp43 (L195F). Similar to what we observed with human USP18, a L195F substitution in mUSP18/Ubp43 impaired its ability to react with the ISG15-VS ABP (Fig. S7*A*) as well as the ISG15-AMC substrate (Fig. S7, *B* and *C* for control of expression). Altogether, these results define a conserved leucine residue in USP18, at position 198 (human) or 195 (mouse), to be required for its activity toward ISG15.

### AlphaFold-guided analysis identifies Tyr363 and Leu198 as crucial for ISG15 binding and USP18 activity

**Figure 6:**
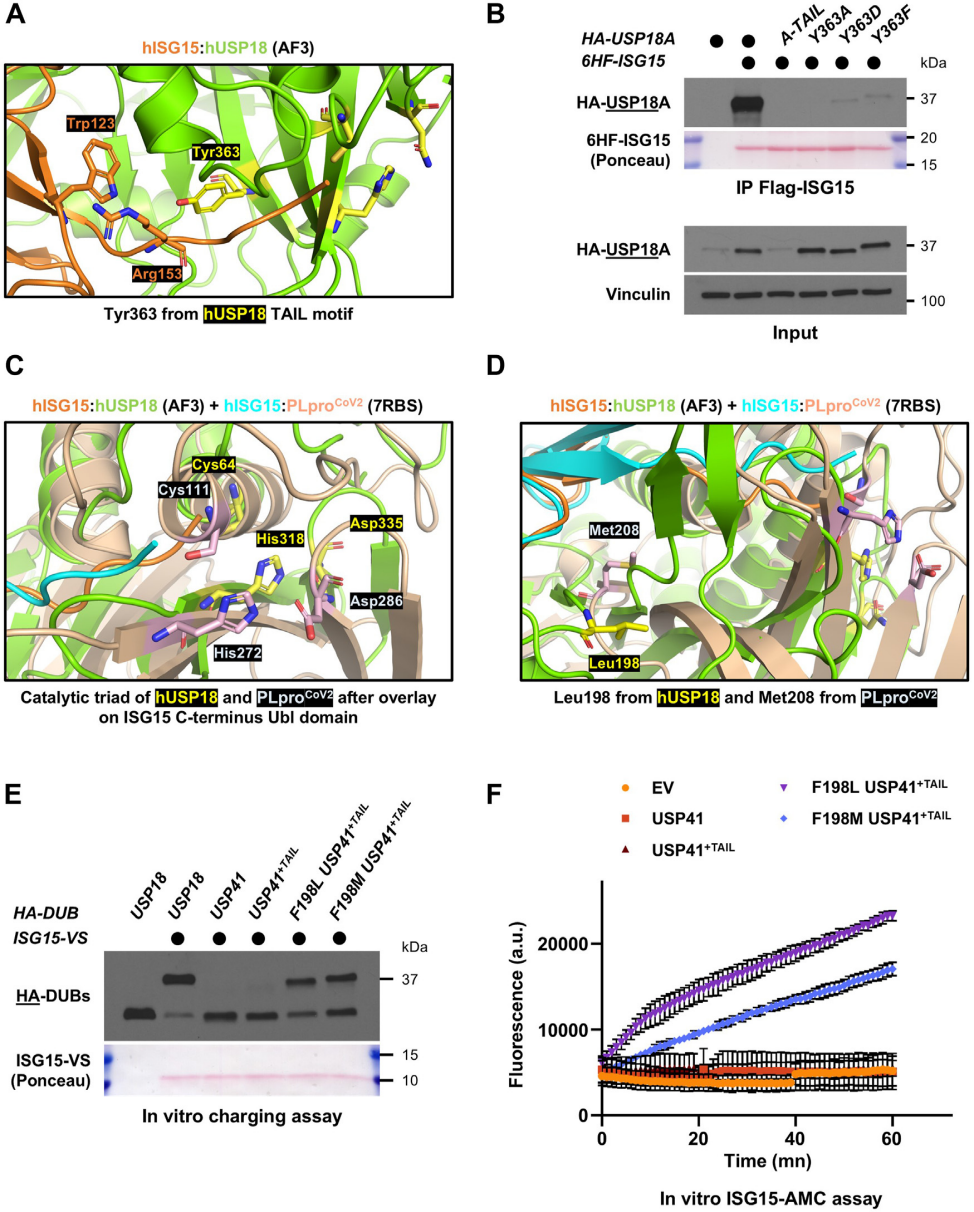
**AlphaFold-guided analysis identifies Tyr363 and Leu198 as crucial for ISG15 binding and USP18 activity**. *A*, close view of Tyr363 from USP18 TAIL motif using AlphaFold 3, and its position respective to Trp123 and Arg153 of hISG15. Residues from the catalytic triad (Cys64, His318, Asn335) are colored in *yellow*. *B*, WT, A-TAIL, or Tyr363 mutant versions of HA-USP18A were ectopically expressed in HEK-293T cells along with 6HF-ISG15. After 24 h, anti-FLAG magnetic beads were used to immunoprecipitate ISG15 from precleared lysates of transfected cells. Immunoblotted antigen is underlined to the *left* of the blots. Representative of >3 independent, biological replicates. *C*, close view of the catalytic triads of hUSP18 and PLpro^CoV2^, by overlaying the AlphaFold structure of hISG15: hUSP18 with the crystal structure of hISG15:PLpro^CoV2^ (PDB identifier: 7RBS) on the C-terminal Ubl domain of ISG15. The catalytic triad of USP18 (Cys64, His318, Asp335) is in *yellow*, and the catalytic triad of PLpro^CoV2^ (Cys111, His272, Asp286) is in *light pink*. *D*, close view of Leu198 from hUSP18 in *yellow* and Met208 from PLpro^CoV2^ in *light pink*, found in a similar space and orientation by overlaying the two structures on ISG15 C-terminal Ubl domain like in (*C*). *E*, the indicated HA-tagged constructs were expressed in HEK-293T cells and immunoprecipitated on anti-HA beads. Immunoprecipitates were mixed with reaction buffer containing ISG15-VS or not, and reaction products were analyzed by SDS-PAGE and Western blot. Representative of >3 independent, biological replicates. *F*, same as in (*E*), except that the purified HA-tagged DUBs were mixed with reaction buffer containing the fluorogenic substrate ISG15-AMC, and fluorescence increase was monitored as previously described. Representative of >3 independent, biological replicates.

Having established that the TAIL motif and Leu198 are important for USP18 binding to ISG15 and enzymatic activity, respectively, we sought to put these findings in a structural context. To do so, we took advantage of AlphaFold to predict the structure (41) of the catalytic domain of human USP18 in complex with human ISG15 (Fig. S8*A*, with the TAIL motif and Leu198 shown in red). Unsurprisingly, the AlphaFold prediction of the human complex showed high structural similarity to that of mUSP18 with mISG15 (PDB ID: 5CHV), with an overall rmsd of 0.60 Å, and recapitulated the key feature that USP18 recognizes ISG15's C-terminal Ubl domain (35), leaving the distal N-terminal Ubl domain available for additional interactions (Fig. S8*A*). Analysis of our AlphaFold structure of the hISG15:hUSP18 complex showed that Tyr363 of USP18, in the TAIL motif, is located within 5 Å of Trp123 and Arg153 on ISG15 (Fig. 6*A*). Interestingly, these residues were shown to mediate hydrophobic interactions between mISG15 and mUSP18/Ubp43 (35). Therefore, we hypothesized that Tyr363 might be involved in mediating the interaction between USP18 and ISG15. To test this, we generated several point mutations of Tyr363 that were assessed for binding to ISG15 by co-immunoprecipitation. Remarkably, mutating Tyr363 into an alanine, aspartic acid, or phenylalanine abolished USP18 binding to ISG15, irrespective of which protein was precipitated (Figs. 6*B* and S8*B*). Accordingly, these Tyr363 mutant versions of USP18 were strongly impaired in their ability to react with the ISG15-VS ABP (Fig. S8*C*). Thus, this supports our model that the TAIL motif contributes to USP18 binding to ISG15 and further suggests that Tyr363 is a critical residue which mediates this interaction.

To better understand the role of Leu198 in mediating USP18 enzymatic activity, we turned our attention to the PLpro protease from SARS-CoV2, the virus responsible for COVID-19 (42). PLpro^CoV2^ is an open reading frame (ORF) located on the non-structural protein 3 (Nsp3) that has been shown to exert its function by acting as both a DUB and a deISGylase (43, 44). Interestingly, and despite low sequence identity, PLpro enzymes from various coronaviruses share common structural and catalytic properties with the family of Ubiquitin-specific proteases (USPs) (45). This is illustrated by overlaying the AlphaFold structure of hISG15:hUSP18 with the recently solved crystal structure of PLpro^CoV2^ in complex with human ISG15 (44) (hISG15:PLpro^CoV2^, PDB ID: 7RBS), through pairwise alignment on the ISG15 molecules. While the structures of both proteases differ significantly (Fig. S8*D*), this analysis also reveals several shared structural features, particularly the highly similar architecture of their catalytic triads (Fig. 6*C*). Key residues on PLpro^CoV2^ were identified to mediate non-covalent interactions with ISG15, including Tyr171, Glu167 and Met208 (44). We identified corresponding residues in our AlphaFold structure of hISG15:hUSP18. Interestingly, Leu198 of USP18 appears to be in a similar space and orientation to Met208 of PLpro^CoV2^, and at the end of a beta strand for both residues (Fig. 6*D*). Because Met208 was described as a key residue for the viral enzyme, and we found that Leu198 was important for USP18 activity, this could suggest that the presence of either a leucine or methionine in that part of the protease could be important for both viral and human mechanisms of deISGylation. To test this hypothesis, we used our USP41^+TAIL^ chimera that is inactive towards ISG15. Because the chimera USP41^+TAIL^ can be activated by mutating this residue to the corresponding Leucine of USP18 (F198L, Fig. 5*C*), we tested whether mutating that phenylalanine to methionine (F198M) would lead to the same activation. We generated a F198M USP41^+TAIL^ chimera and assessed its activity using the ISG15-VS and ISG15-AMC assays. Strikingly, the F198M mutation activated the chimera USP41^+TAIL^ to a similar extent as the F198L replacement (Figs 6, *E* and *F*, and S8*E* for control of expression). This suggests that a leucine or methionine in that region can support the enzymatic activity of deISGylating enzymes.

Altogether, by combining analysis of the AlphaFold structure of hISG15:hUSP18 with multiple biochemical assays, we propose that both Leu198 and Tyr363 of the TAIL motif act as functional surfaces that contribute to enzymatic activity and ISG15 binding, respectively.

## Discussion

USP18 is part of the USP family of DUBs, which is the largest subfamily of ubiquitin cysteine proteases (46). Despite its name, and some reports suggesting that it regulates ubiquitination of a few substrates (47, 48), the most well-documented function of USP18 is to cleave ISG15 from intracellular proteins (33). Moreover, human USP18 can non-covalently bind to ISG15 with very high affinity (34), yet the molecular basis for USP18's deISGylating activity remains unclear.

To find new molecular determinants of USP18 for ISG15, we performed a comparative analysis with USP41, a close paralog of USP18 with high sequence homology (Fig. 2*A*). Despite being related, USP41 does not act as an ISG15 protease (Fig. 2). However, the very high sequence similarity between the catalytic domains of the two gene products (>97%) offered us the unique opportunity to decipher the basis for their different enzymatic activity, with the goal of characterizing new molecular determinants of USP18's specificity for ISG15. By combining molecular and biochemical approaches, we identified a stretch of six evolutionary conserved residues on USP18 C-terminus that we called the TAIL motif, which appears critical for binding of USP18 to ISG15 (Fig. 4). Using AlphaFold-guided structural analysis in combination with biochemical assays, we provide experimental evidence that Tyr363 in the TAIL motif is a key residue mediating binding of USP18 to ISG15 (Fig. 6).

The importance of Tyr363 and the TAIL motif is particularly interesting when considering the various mechanisms of action of USP18. In addition to its role in deconjugating ISG15 from proteins in an enzymatic-dependent manner, mUSP18/Ubp43 can also negatively regulate interferon signaling in an enzymatic-independent manner, by competing with JAK1 for binding to the interferon receptor IFNAR2 (31). Interestingly, it was shown that the C-terminus of USP18 mediates the interaction with IFNAR2, and the authors suggested that the residues within the last 50 amino acids of USP18 might be critical for this interaction. Remarkably, Richer *et al.* reported that a missense mutation in the TAIL motif of mUSP18/Ubp43 severely impacted mice susceptibility to *Salmonella typhimurium* infection (49). The authors identified a loss-of-function mutation within the TAIL motif of mUSP18/Ubp43, where replacing Leu361 (Leu364 in human USP18) to phenylalanine conferred increased susceptibility to *S. typhimurium* (49). It is tempting to speculate that different residues within the TAIL motif might dictate the function of USP18 in different processes. For instance, Leu364 could coordinate the regulation of IFNAR2 signaling while Tyr363 would facilitate ISG15 binding. However, additional studies will be needed to address the exact contribution of these different residues within the TAIL motif in mediating the different mechanisms of action of USP18.

Although the TAIL motif appears necessary for USP18 binding to ISG15, it is not sufficient to turn USP41 catalytic domain into an ISG15 protease, since our chimeric construct USP41^+TAIL^ was still inactive towards ISG15. By performing amino acids swapping, we demonstrate that Leu198 of USP18 is required for enzymatic activity, and likewise, that it is sufficient to turn the inactive USP41^+TAIL^ into an active ISG15 protease (Figs. 5 and S6). Accordingly, mutating Leu198 (Leu195 in mouse) to phenylalanine strongly reduced enzymatic activity of both USP18 and mUSP18/Ubp43 (Figs. 5, *E* and *F*, S6, *E* and *F*), highlighting the importance of that residue for enzymatic activity. To put these findings in a structural context, we took advantage of AlphaFold to generate the structure (41) of human USP18 catalytic domain in complex with ISG15 and compared it to the crystal structure of the COVID19 protease PLpro^CoV2^, a DUB-like protease which possesses deISGylating activity (Fig. 6*C*). Interestingly, this analysis revealed that Leu198 of USP18 appears to be in a similar space and orientation as Met208 of PLpro^CoV2^, which was described as a key residue for the function of the viral protease (44). Importantly, our data shows that we can turn the inactive USP41^+TAIL^ into a deISGylating enzyme by also introducing that methionine (Fig. 6, *E* and *F*). Therefore, it is tempting to speculate that the presence of a small hydrophobic residue in that part of the protease is a common feature of both human and viral deISGylating enzymes. Of note, this feature was not reported when the crystal structure of the catalytic domain of mUSP18/Ubp43 in complex with mouse ISG15 was solved (35, 50). The main finding of that study explaining USP18's selectivity was the identification of the hydrophobic domain termed IBB1, which forms a patch made of four residues (Ala138, Leu142, Ser192 and His251) that appears crucial for interacting with a hydrophobic region unique to ISG15 located on its C-terminus Ubl domain. Mutation of all four IBB1 residues impair USP18 binding to ISG15 (Fig. 4*G*), making it clear that these amino acids participate in ISG15 recognition. However, IBB1 is present on the catalytic domain of USP41 and yet, our chimera USP41^+TAIL^ did not show any enzymatic activity (Fig. S5*A*), suggesting that IBB1 alone, in the context of a DUB domain, is not sufficient to explain USP18's activity toward ISG15.

Our findings that both Leu198 and the TAIL motif are important for USP18 function were facilitated by performing a comparative analysis with its paralog USP41, that we found to be a new USP18-interacting partner, even though the function of their interaction remains to be elucidated. Interestingly, over the course of our study, USP41 went from being annotated as a protein-coding gene in databases to now being a pseudogene. The notion that USP41 could be a pseudogene was suggested in a study done by the Pellegrini lab, in which the authors argued that the USP41 gene lacks both 5′ and 3′ UTRs, on top of not finding evidence of USP41 transcripts in human tissues (51). However, an early study which performed whole transcriptomic profiling by RNA-sequencing (RNA-seq) of macrophages stimulated with LPS found USP41 to be one of the most upregulated genes following LPS activation (52). Furthermore, we were able to detect protein peptides specific to USP41 in our mass-spectrometry experiment (Fig. 1*C* and S1*A*), indicating that the mechanisms by which USP41 gets transcribed and translated require further investigation. Another possibility explaining the scarcity of evidence regarding the existence of USP41 could lie in its expression pattern, and we hypothesize that USP41 expression might also be limited to certain tissues, which has been observed in an early study that cloned and analyzed 22 USPs, including USP41 (53). This will represent the basis for future work aimed at delineating the function, mechanisms of action, and expression pattern of USP41.

The high sequence homology of USP41 with USP18, coupled with its inability to regulate deISGylation, offered us a unique opportunity to gain insights into the molecular determinants of USP18 specificity towards ISG15. Therefore, our work highlights the power of performing comparative analysis between paralog genes, like USP18 and USP41, to identify the biochemical basis for their differences and illuminate unique features of protein function. Importantly, USP18 has been implicated in diseases, thus understanding the mechanisms governing its catalytic activity might aid in the future development of inhibitors to impact disease progression.

## Experimental procedures

### Mammalian cell culture, transfection, cell lysis, antibodies, and reagents

HEK-293T cells were obtained from ATCC and grown in DMEM complete medium (Gibco, Cat# 11–965–092) supplemented with 10% fetal bovine serum (Atlanta Biologicals). All DNA transfection experiments were done using PolyJet In Vitro DNA Transfection Reagent (SignaGen Laboratories) according to the manufacturer's instructions and subsequently cultured for 24 to 48 h prior to analysis. Samples for protein analysis by immunoblot were lysed in NETN buffer [20 mM Tris-Cl (pH 8.0), 100 mM NaCl, 0.5 mM EDTA and 0.5% (v/v) Nonidet P-40 (NP-40)] supplemented with 2 μg/ml pepstatin, 2 μg/ml apoprotinin, 10 μg/ml leupeptin, 1 mM AEBSF [4-(2 Aminoethyl) benzenesulfonyl fluoride], and 1 mM Na_3_VO_4_. Lysis was performed on ice for ∼10 min with occasional vortexing, lysates were then spun down at 14,000 rpm for 10 mn before determining protein concentration using Bradford reagent (Bio-Rad).

Standard immunoblotting procedures were followed. A list of reagents and antibodies that were used in this study, including the concentrations at which they were used, is available in Table S3. All antibodies were diluted in 5% nonfat dried milk [diluted in Tris buffered saline, 0.05% tween-20 (TBST)], incubated for 1 h at room temperature or overnight at 4 °C, and detected using HRP conjugated secondary antibodies (Jackson Immuno Research Laboratories Inc; 1:5000 dilutions).

### ISG15 immunoprecipitation and mass spectrometry analysis

#### Sample preparation

For mapping PTMs on USP18 by mass spectrometry after ISG15 immunoprecipitation, three sets of 10x 100-mm dishes of HEK-293T cells were transfected with a total of five ug per plate (2.5 μg of untagged USP18, either alone or with 2.5 μg of 6HF-ISG15) and using PolyJet reagent according to the manufacturer's instructions. Cells were grown for 48 h, washed with PBS, and harvested in PBS then centrifuged at 1,500*g* for 5 mn. Cell pellets were lysed in NETN lysis buffer supplemented with protease and phosphatase inhibitors, as described above. Lysates were also snap frozen twice in liquid nitrogen, and then lysates were clarified by centrifugation at 14,000 rpm for 10 min at 4 °C. Protein concentration was normalized using Bradford reagent. Samples were immunoprecipitated using EZview Red anti-FLAG M2 affinity gel (Millipore Sigma) for 2 h at 4 °C on a rotary shaker. Following IP, samples were washed 3x using NETN buffer followed by three washes using dPBS (Gibco, Cat# 14190144). Samples were resuspended in 2X Laemmli buffer, separated by SDS-PAGE on a pre-cast 4 to 15% Criterion Protein Gel (Bio-Rad Laboratories, Cat#5671084), and the gel was stained using QC Colloidal Coomassie Stain (Bio-Rad) according to the manufacturer's instructions. The bands corresponding to USP18 that co-precipitated with ISG were excised into 1 mm cubes, and destained for 20 min. Gel bands were washed with ACN, reduced with 10 mM DTT, alkylated with 100 mM iodoacetamide, and digested in-gel with 0.5 ug/uL trypsin overnight at 37 °C. Peptides were extracted with ACN, desalted with Pierce C18 spin columns (ThermoFischer Scientific, Catalog # 69705) and dried *via* lyophilization. Peptide samples were stored at −80 °C until further analysis.

#### LC-MS/MS analysis

The peptide samples were analyzed by LC/MS/MS using an Easy nLC 1200 coupled to a QExactive HF mass spectrometer (ThermoFischer Scientific). Samples were injected onto an EasySpray column (75 μm id × 25 cm, 2.0 μm particle size) and separated over a 45 min method. The gradient for separation consisted of 5 to 45% mobile phase B at a 250 nl/min flow rate, where mobile phase A was 0.1% formic acid in water and mobile phase B consisted of 0.1% formic acid in 80% ACN. The QExactive HF was operated in data-dependent mode where the 15 most intense precursors were selected for subsequent fragmentation. Resolution for the precursor scan (m/z 350–1700) was set to 120,000 with a target value of 3 × 106 ions. MS/MS scans resolution was set to 15,000 with a target value of 1 × 105 ions. The normalized collision energy was set to 27% for HCD. Dynamic exclusion was set to 30 s, peptide match was set to preferred, and precursors with unknown charge or a charge state of one and ≥ 6 were excluded.

#### Data analysis

Raw data files were processed using Sequest HT within Proteome Discoverer version 2.5 (ThermoFischer Scientific). Data were searched against a reviewed Uniprot human database (downloaded in February 2020), appended with a common contaminants database (MaxQuant, 245 sequences). The following parameters were used to identify tryptic peptides for protein identification: 10 ppm precursor ion mass tolerance; 0.02 Da product ion mass tolerance; up to two missed trypsin cleavage sites; (C) carbamidomethylation was set as a fixed modification; (M) oxidation, (K) Acetylation, (K) Ubiquitination, and (S/T/Y) phosphorylation were set as variable modifications. Peptide false discovery rates (FDR) were calculated by the Percolator node using a decoy database search and data were filtered using a 1% FDR cutoff. MS/MS spectra corresponding to unique peptides mapping to USP41 (identified as the co-precipitated protein) were annotated using IPSA (54).

### Data availability

Processed proteomics data files are available as supplemental tables. The mass spectrometry proteomics data have been deposited to the ProteomeXchange Consortium *via* the PRIDE partner repository with the dataset identifier PXD058618.

### Reviewer access details

Log in to the PRIDE website using the following details.

### Project accession

PXD058618.

### Token

g7L5sEMl8xHS.

Alternatively, reviewers can access the dataset by logging in to the PRIDE website using the following account details.

### Username

reviewer_pxd058618@ebi.ac.uk.

### Password

9VyZma22Nujw.

### Molecular biology

USP41 was synthesized by Integrated DNA Technologies, and its sequence can be found in Supplementary Document 1 (gBlocks and protein sequences). FLAG-UbcM8, His_6_-mISG15, and HA-mUbp43 were gifts from Dr Dong-Er Zhang (Addgene plasmid #12440, #12445, and #12454, respectively), while FLAG-HA-USP18 was a gift from Dr Wade Harper (Addgene plasmid #22572). The ISG15 E1 (UBA7) and E2 (UbcH8) enzymes were kind gifts of Dr Robert Krug (University of Texas). These plasmids served as templates to generate all the constructs used in this study, which were subcloned using restriction enzyme-based cloning into pcDNA3.1(+), a kind gift from Dr David Allison. PCR amplification of DNA was performed using Q5 High Fidelity DNA polymerase purchased from New England Biolabs. All PCR primers contained the appropriate restriction sites, followed by a Kozak sequence, an appropriate N-terminal HA, Myc or V5 tag, and a di-glycine linker (see Table S3 for details about plasmids and primers). All primers for site-directed mutagenesis were designed by using the NEBaseChanger website from New England Biolabs (https://nebasechangerv1.neb.com/) and following their protocol. To engineer the chimeric USP41^+TAIL^ constructs, we took advantage of the EcoRI restriction site present at position 787 of both genes. First, we PCR amplified a USP18 fragment, which started at the EcoRI site and covered the rest of USP18 sequence, while adding an XhoI site after the stop codon. Then, USP41 constructs were digested with EcoRI and XhoI, and ligation was done with the USP18 fragment. All constructs were verified by sequencing.

### Immunoprecipitation

Immuno-precipitation experiments (IP) were performed essentially as described previously (55). Briefly, HEK-293T cells were transfected in 60-mm dishes with a total of 2.5 μg of DNA constructs for 24 to 48 h. Cells were washed and scrapped in PBS then lysed for 10 min at 4 °C with occasional vortexing in NETN buffer supplemented with protease inhibitors. Cell debris was removed by centrifugation at 14,000 rpm for 10 min at 4 °C. Inputs were prepared with the clarified lysate, then anti-FLAG, anti-HA or anti-Myc beads (20 μl per IP, all from Millipore Sigma) pre-washed with lysis buffer were mixed with the remaining lysate and incubated on a rotary shaker for 1h to 1h30 at 4 °C. Beads were washed with lysis buffer four times, resuspended in 25 ul of 2X sample buffer (60 mM Tris pH 6.8, 2% SDS, 10% glycerol, 100 mM DTT and 0.1% Bromophenol Blue) then incubated at 95 °C for 5 min before SDS-PAGE and Western blot analysis.

### Overexpression system to study ISGylation in HEK-293T cells

Generation of broad protein ISGylation in cells was performed by using an overexpression approach in HEK-293T cells based on a previously described protocol (56), with minor changes. Briefly, HEK-293T cells were first seeded in 60-mm dishes, at approximately three million cells per dish. The day after, cells were transfected with a mix of 0.375 μg of V5-UBA7 (E1), 0.375 μg of V5-UbcH8 (E2), and 0.75 μg of 6HF-ISG15. Where indicated, 0.75 μg of the indicated DUBs constructs was also included; otherwise, empty pcDNA3.1(+) was used to normalize DNA concentration. After 24 h of transfection, cells were washed once with PBS, harvested in PBS, and spun down at 1,000*g* for 3 mn. Cell pellets were resuspended in 200 μl of phosphate lysis buffer (50 mM NaH_2_PO_4_, 150 mM NaCl, 1% Tween-20, 5% Glycerol, pH 8.0) supplemented with 2 μg/ml pepstatin, 2 μg/ml apoprotinin, 10 μg/ml leupeptin, 1 mM AEBSF [4-(2 Aminoethyl) benzenesulfonyl fluoride], and 1 mM Na_3_VO_4_. Lysis was performed on ice for ∼10 min with occasional vortexing, debris was centrifuged at 14,000 rpm for 10 mn before, and protein concentration was calculated using Bradford reagent. For each condition, 20 to 30 μg of total protein was separated by SDS-PAGE on a pre-cast 4 to 15% Criterion Protein Gel (Bio-Rad Laboratories, Cat#5671084) followed by an overnight transfer and Western blot analysis.

### *In vitro* charging assay of DUBs with the ISG15-VS activity-based probe

Charging of USP18, USP41, Ubp43, and all other variants, with the ISG15-VS activity-based probe (ABP) was done in a semi-pure way where DUBs were first expressed and immunoprecipitated from HEK-293T cells then used for *in vitro* assays. Typically, a 60-mm or 100-mm dish of confluent HEK-293T cells was transfected as described using 2.5 μg of DNA. After 24 to 48 h of expression, cells were washed once with PBS, harvested in 1 ml of PBS then centrifuged at 1,000*g* for 3 mn, and cells pellets were either stored at −80 °C or lysed immediately. Cell lysis was done using phosphate lysis buffer supplemented with 2 μg/ml pepstatin, 1 mM AEBSF [4-(2 Aminoethyl) benzenesulfonyl fluoride], 1 mM Na_3_VO_4_ and 10 mM DTT. Cell lysis was done for 10 mn on ice with occasional vortexing, then cell debris was removed by centrifugation at 14,000 rpm for 10 mn at 4 °C. For each plate of cells expressing the desired construct, which typically gave 1 to 5 mg of protein lysate (for 60- or 100-mm dishes, respectively), the DUB of interest was immunoprecipitated using 30 μl of anti-HA or anti-Myc beads for 1h at 4 °C. Beads were washed once with lysis buffer (4 °C), once with PBS (RT), then resuspended in DUB activation buffer (pre warmed at 37 °C) and split into two tubes. Beads were spun down, resuspended in 10 μl of DUB activation buffer (25 mM Tris pH 7.5, 150 mM NaCl and 10 mM DTT, prewarmed at 37 °C), incubated for 10 mn at 37 °C then mixed at a 1:1 ratio with DUB activation buffer containing 10 μM of ISG15-VS or no ABP as a negative control (buffer only). Reactions were incubated for 2 h at 37 °C, quenched by adding 10 μl of 4X Laemmli buffer (62.5 mM Tris pH 6.7, 2% SDS, 10% glycerol, 100 mM DTT and 0.1% Bromophenol Blue) and separated by SDS-PAGE. Charging of DUBs with ISG15-VS was detected by western blotting the tag fused to the DUB.

### *In vitro* DUB assays using the fluorogenic substrate ISG15-AMC

The assay was conducted in a very similar way to the charging assay with ISG15-VS. Briefly, the indicated DUB constructs were expressed and purified from HEK-293T cells exactly as described above. Following IP of the DUBs, beads were washed once with lysis buffer (4 °C), once with PBS (RT), and once with DUB buffer (50 mM Tris pH 7.5, 50 mM NaCl, 100 mg/ml BSA, and 10 mM DTT, prewarmed at 37 °C). Following the last wash, beads were resuspended in 40 μl of DUB buffer and then split into three wells of a 384-well white flat plate (Corning, Cat# 3826BC), with 10 μl of immunoprecipitates per well. Following a 10 mn incubation at RT, each well was mixed with 10 μl of ISG15-AMC (R&D Systems, Cat# UL-553) that was diluted to 2 μM in DUB buffer. Release of AMC fluorescence was monitored using a BioTek Cytation 5 (Agilent Technologies) with an excitation wavelength of 380 nm and an emission wavelength of 460 nm. Reactions were carried out for either 45 or 60 mn, and readings were done every mn. Raw measurements were collected and presented as plots that were generated using GraphPad Prism.

### Size exclusion chromatography

HEK-293T cells were seeded in 100-mm dishes and transfected with either HA-USP18 FL or HA-USP18 ΔC-term as described before. Cells were lysed 48 h later using phosphate lysis buffer and lysates were clarified *via* centrifugation. The resulting lysates, containing ∼ 5 mg of total protein, were then analyzed by gel filtration chromatography. Protein complexes from both lysates were separated using a size exclusion column (Superose 6 Increase, 10/300, Cytiva Life Sciences) that had been pre-equilibrated using phosphate lysis buffer. During separation, 0.5 ml fractions were collected and later analyzed by SDS-PAGE and immunoblot, or SDS-PAGE and SYPRO Stain (ThermoFischer Scientific, Catalog #S11791) following the manufacturer's instructions.

### Structure generation using AlphaFold three

We took advantage of AlphaFold 3 (https://alphafoldserver.com) to generate and predict the structure (41) of hUSP18:hISG15. The sequences that were used can be accessed in the Supplementary Document one attached to this manuscript. Afterward, the top-ranked structure was selected then visualized using PyMOL. The PBD identifiers of the hISG15:PLpro^CoV2^ and mISG15:Ubp43 complexes solved by X-ray crystallography are 7RBS and 5CHV, respectively.

## Data availability

All data supporting the main findings of our study are in the manuscript and in the supporting information. All plasmids generated and described here are available upon request. Finally, the raw mass spectrometry data have been deposited to the ProteomeXchange Consortium *via* the PRIDE partner repository (as described in Experimental procedures).

## Supporting information

This article contains supporting information.

## Conflict of interest

The authors declare that they have no conflicts of interest with the contents of this article.
