# Supplementary material for: Mechanisms of USP18 specificity toward ISG15 revealed by paralog sequence analysis comparison
Source: J Biol Chem. 2025 May 26;301(7):110288. doi: 10.1016/j.jbc.2025.110288 (PMC12221285; doi:10.1016/j.jbc.2025.110288)
Supplement: Supplementary Figures [file mmc1.pdf]

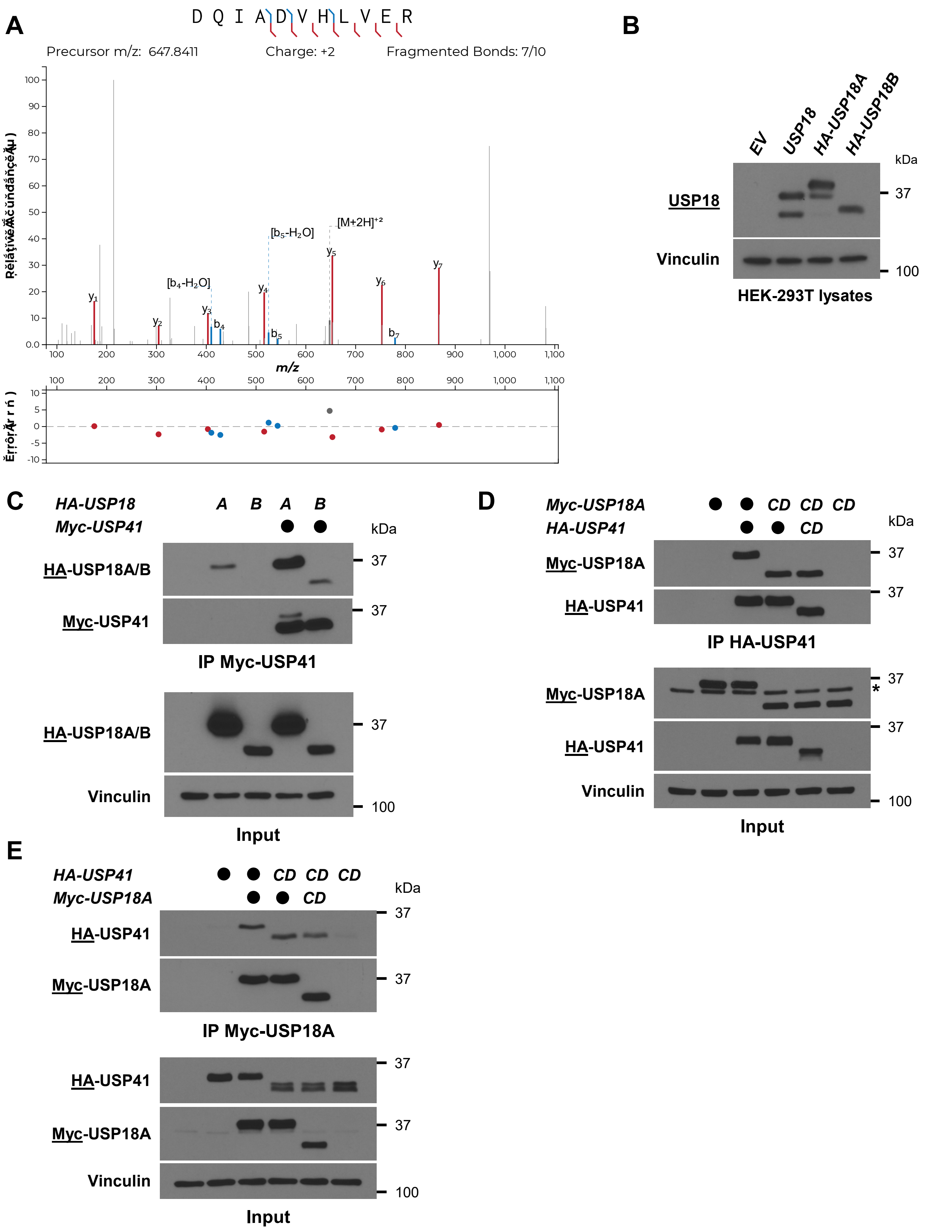

Supplementary Figure 1. USP18 interacts with USP41.

Supplementary Figure 1. USP18 interacts with USP41.

(a) MS/MS spectrum of the doubly charged ion (m/z 647.8411) corresponding to USP41 tryptic peptide DQIADVHLVER.

(b) HEK-293T cells were transfected with the indicated expression constructs and cell lysates were probed for USP18 using a monoclonal antibody raised against USP18 N-terminus (underlined). EV, empty vector. Representative of 2 independent, biological replicates.

(c) Myc-USP41 and HA-USP18 (both isoforms) were ectopically expressed in HEK-293T cells. After 24h, precleared lysates of transfected cells were used to immunoprecipitate USP41 on anti-Myc beads. Immunoblotted antigen is underlined to the left of blots. Representative of >3 independent, biological replicates.

(d) Same as in (c) except that USP18A was used, as well as the catalytic domains (CD) of both USP41 and USP18A (USP41 CD and USP18A CD, respectively). Representative of 2 independent, biological replicates.

(e) Same as in (d) except that HA-USP41 was immunoprecipitated. Asterisk indicates a non-specific band reacting with the Myc antibody. Representative of 2 independent, biological replicates.

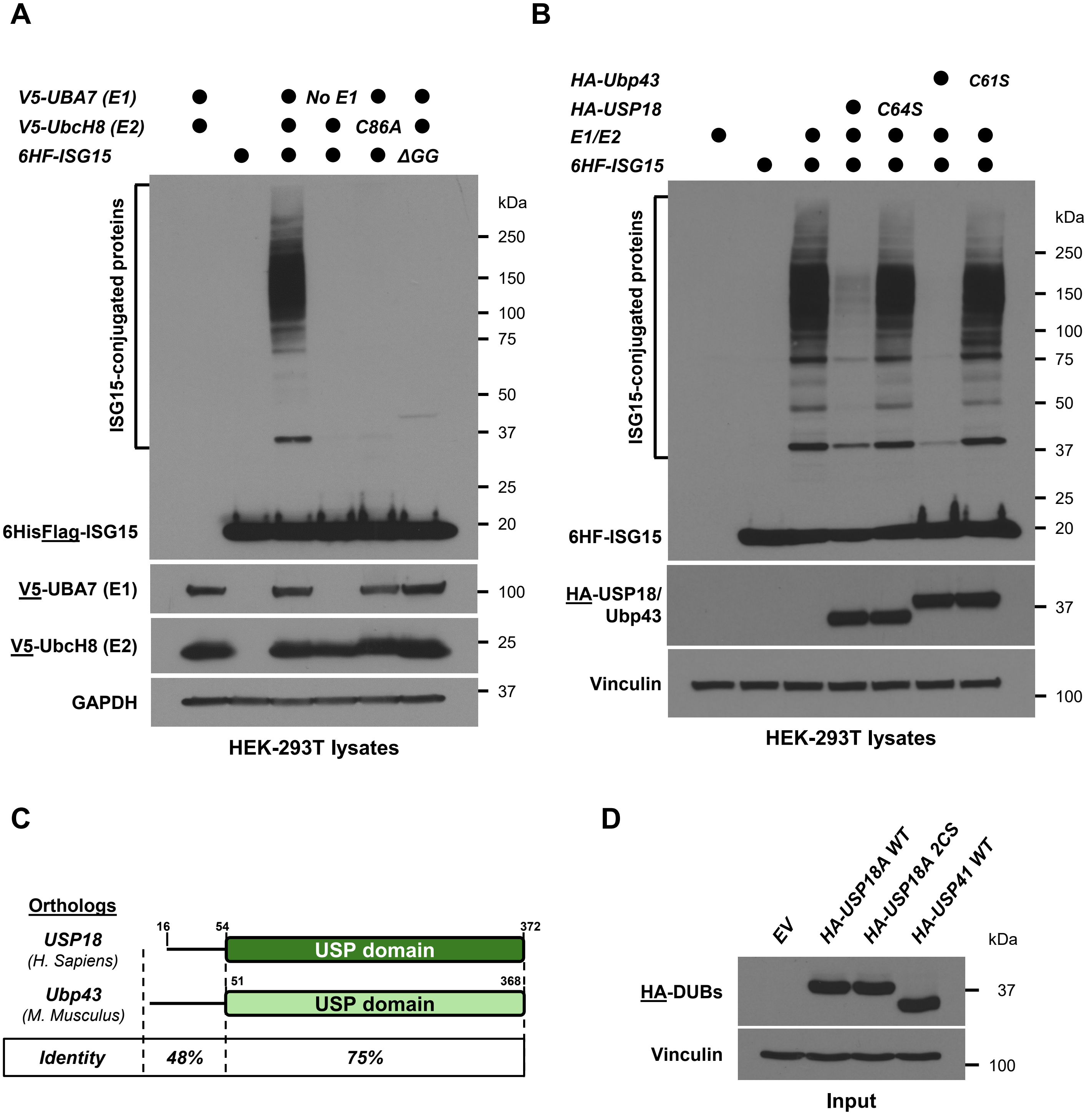

Supplementary Figure 2. Overexpression system in HEK-293T to study protein ISGylation.

Supplementary Figure 2. Overexpression system in HEK-293T to study protein ISGylation.

(a) HEK-293T cells were transiently transfected with 6His-Flag-ISG15 (6HF-ISG15) in combination with vectors encoding the ISG15 E1 activating enzyme (V5-UBA7) and the ISG15 E2 conjugating enzyme (V5-UbcH8). As negative controls, no E1, a catalytically inactive form of UbcH8 (C86A) or a non-conjugatable form of ISG15 (ΔGG) were used. Cell lysates were prepared 24 hours later and protein ISGylation was analyzed by western blotting using the indicated antibodies. Representative of >3 independent, biological replicates.

(b) Same as in Figure 2B, except that WT or catalytically inactive (C61S) versions of HA-Ubp43 were also included. Representative of 2 independent, biological replicates.

(c) Cartoon showing the organization and sequence identity of USP18 and its mouse ortholog mUSP18/Ubp43.

(d) HEK-293T lysates from cells transfected with the indicated constructs were separated by SDS-PAGE and analyzed by western blotting using the indicated antibodies. This serves as a control for the experiment presented in Figure 2D.

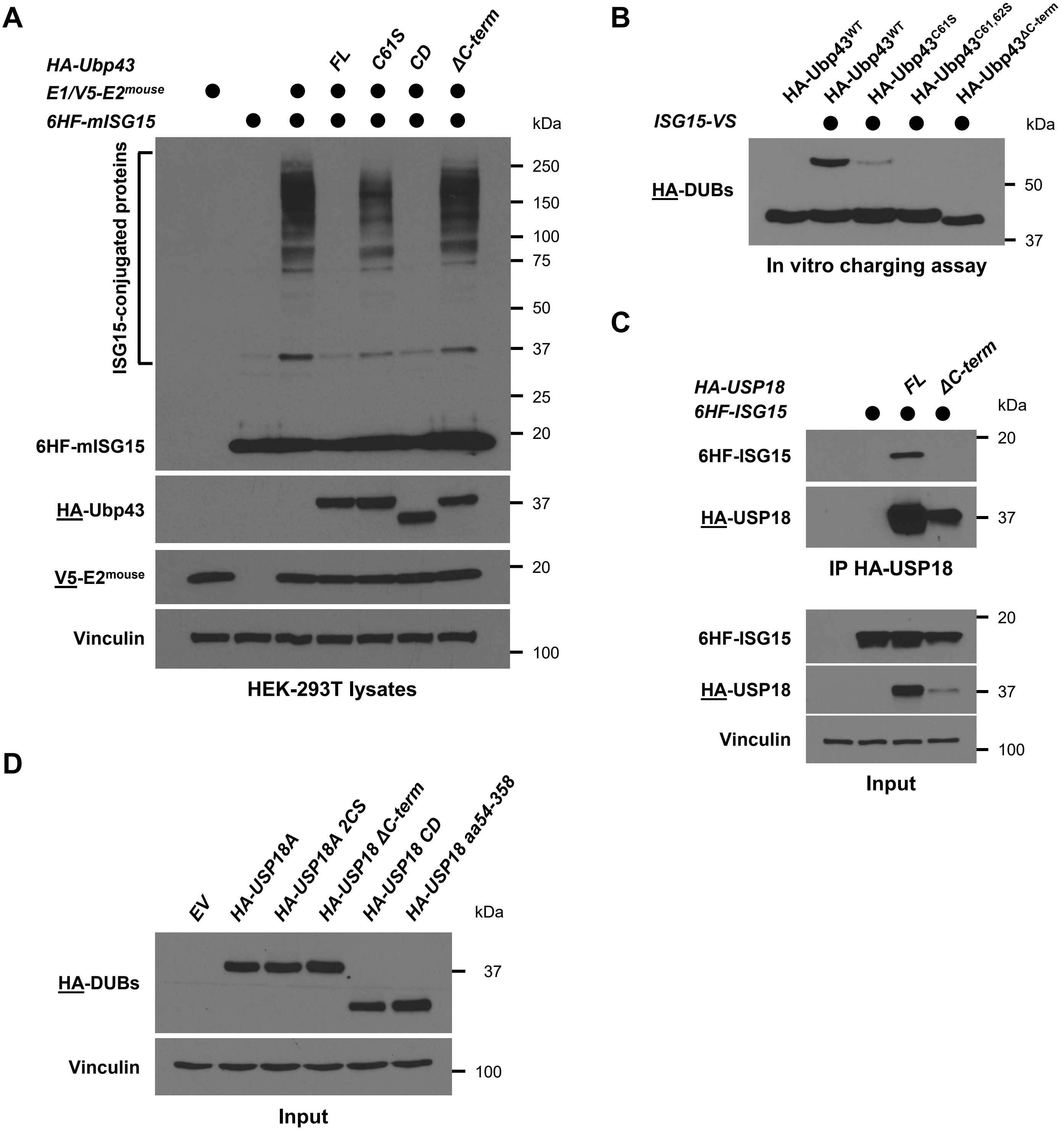

Supplementary Figure 3. The C-terminus of Ubp43 is also necessary for its enzymatic activity.

Supplementary Figure 3. The C-terminus of Ubp43 is also necessary for its enzymatic activity.

(a) Protein ISGylation in HEK-293T cells was reconstituted by transfection of the mice version of the ISG15 machinery (E1/E2^{mouse}/mISG15) and where indicated, HA-tagged Ubp43 constructs were co-transfected. After 24h, lysates of transfected cells were prepared then analyzed by SDS-PAGE and western blot. Immunoblotted antigen is underlined to the left of blots. Representative of 2 independent, biological replicates.

(b) The indicated HA-tagged Ubp43 constructs were ectopically expressed in HEK-293T cells and after 24 h, precleared lysates of transfected cells were used to immunoprecipitate Ubp43 on anti-HA beads. Immunoprecipitates were mixed with reaction buffer containing ISG15-VS or not, and reaction products were analyzed by SDS-PAGE and western blot. Representative of >3 independent, biological replicates.

(c) 6HF-ISG15 was ectopically expressed in HEK-293T cells, either alone or with HA-USP18 FL or HA-USP18 ΔC-term. After 24h, precleared lysates of transfected cells were used to immunoprecipitate USP18 FL or ΔC-term on anti-HA beads. Immunoblotted antigen is underlined to the left of blots. Representative of 2 independent, biological replicates.

(d) Cell lysates of HEK-293T transfected with the indicated HA-tagged USP18 constructs were separated by SDS-PAGE and analyzed by western blotting. This serves as a control for the experiment presented in Figure 3C.

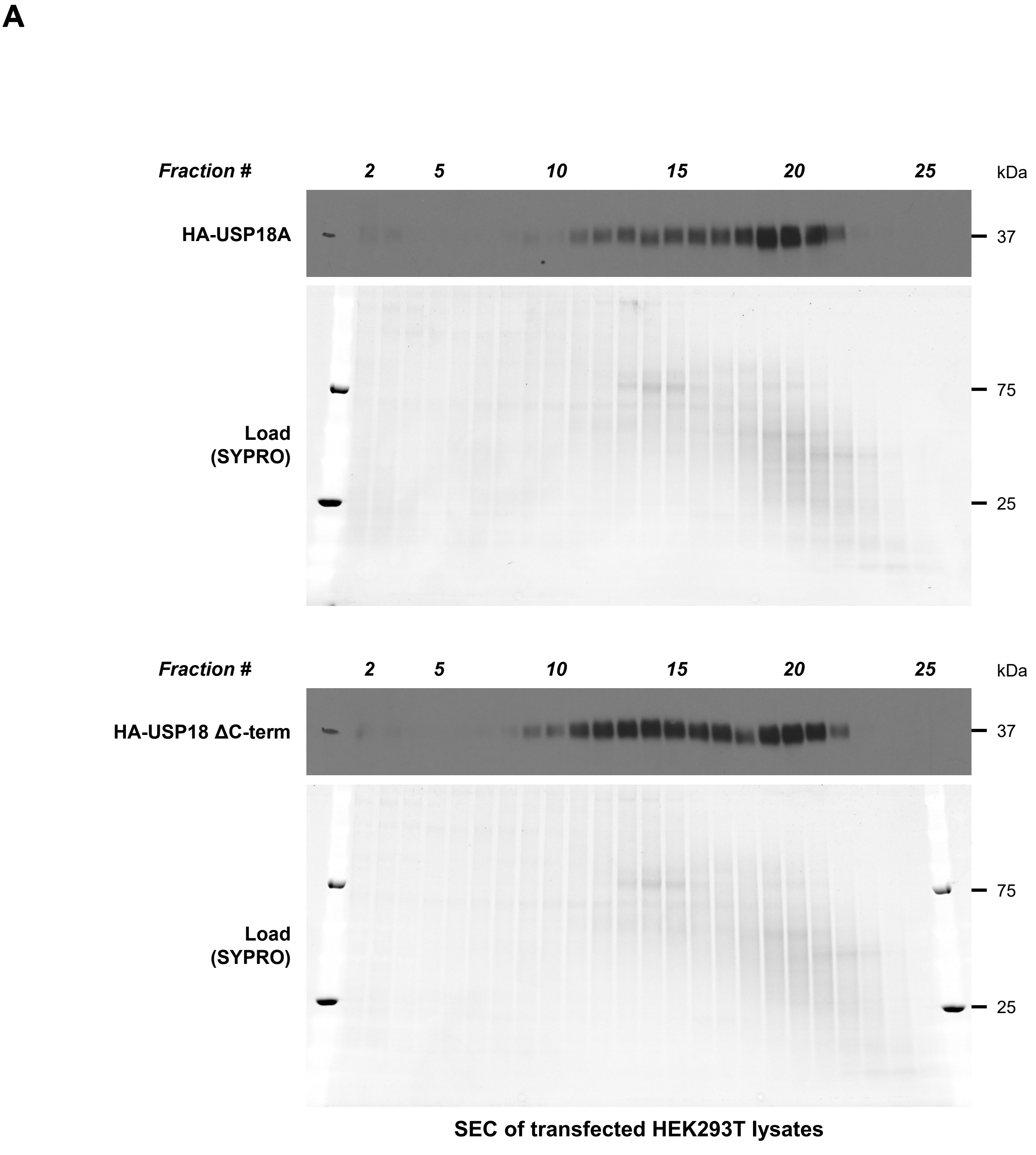

Supplementary Figure 4. Size exclusion chromatography profiles of USP18 and ΔC-term mutant.

Supplementary Figure 4. Size exclusion chromatography profiles of USP18 and ΔC-term mutant.

(a) The indicated HA-tagged USP18 constructs were ectopically expressed in HEK-293T cells, and after 48h, cell lysates were analyzed by size exclusion chromatography (SEC) using a Superose 6 column. SEC fractions were separated by SDS-PAGE and analyzed by western blot as well as SYPRO stain. Representative of 2 independent, biological replicates.

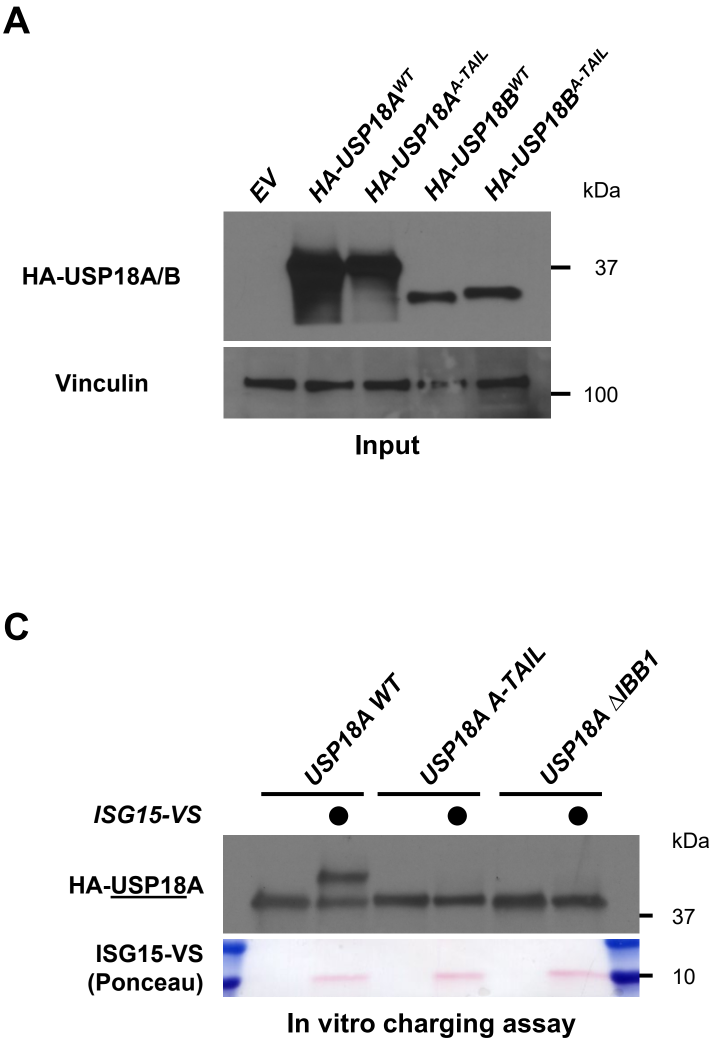

Supplementary Figure 5. The TAIL motif is an important determinant of USP18 binding to ISG15.

Supplementary Figure 5. The TAIL motif is an important determinant of USP18 binding to ISG15.

(a) Cell lysates of HEK-293T transfected with the indicated HA-tagged USP18 constructs were separated by SDS-PAGE and analyzed by western blotting. This serves as a control for the experiment presented in Figure 4D.

(b) WT or A-TAIL versions of HA-USP18A were ectopically expressed in HEK-293T cells, either alone or with 6HF-ISG15. After 24h, precleared lysates of transfected cells were used to immunoprecipitate ISG15 on anti-Flag beads. Immunoblotted antigen is underlined to the left of blots. Representative of >3 independent, biological replicates.

(c) The indicated HA-tagged USP18A constructs were purified from HEK-293T cells on anti-HA beads 24h after transfection. HA-USP18 immunoprecipitates were mixed with reaction buffer containing ISG15-VS or not, and reaction products were analyzed by SDS-PAGE and western blot. Representative of 2 independent, biological replicates.

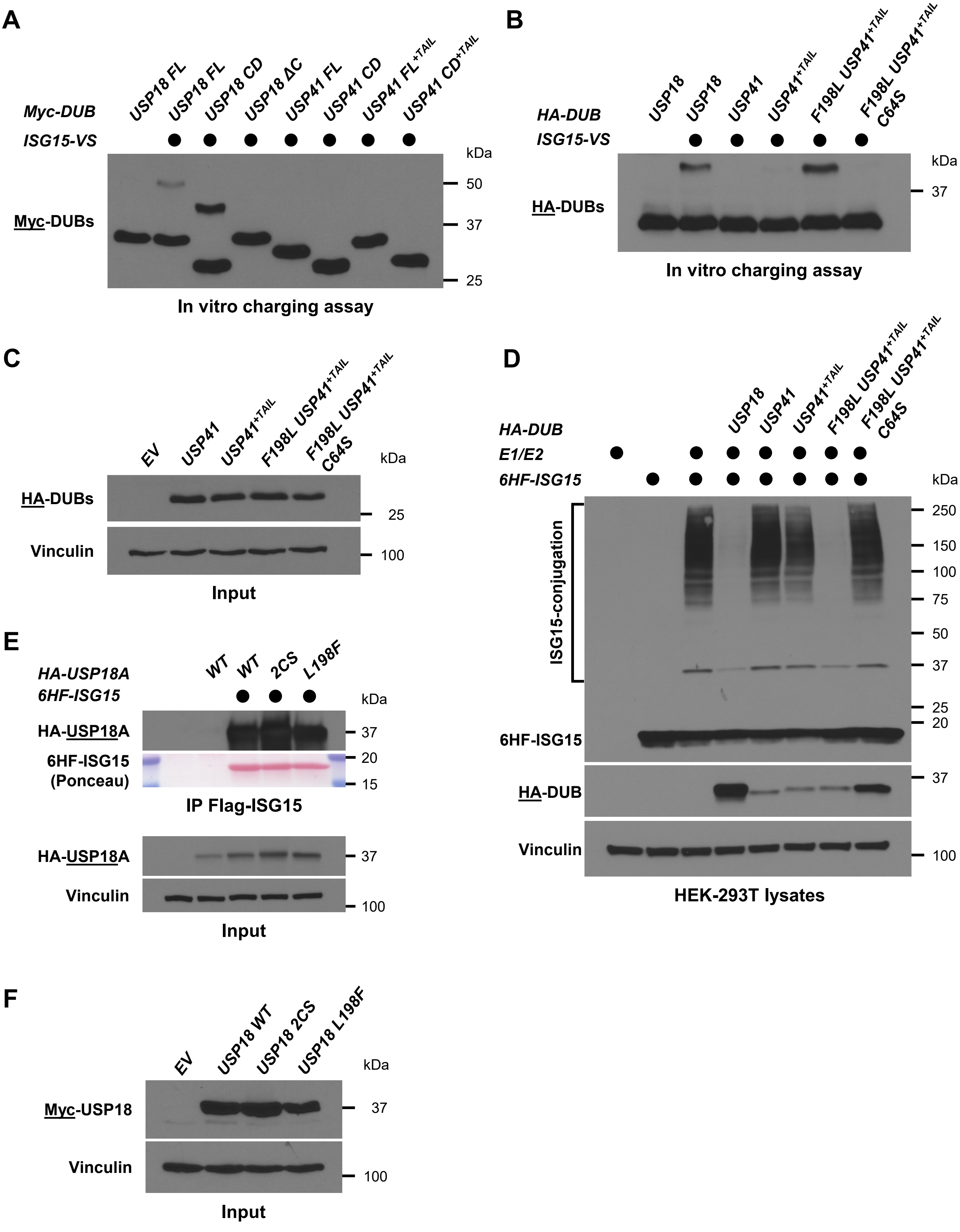

Supplementary Figure 6. Leu198 of USP18 is important for its enzymatic activity.

Supplementary Figure 6. Leu198 of USP18 is important for its enzymatic activity.

(a) The indicated Myc-tagged constructs were ectopically expressed in HEK-293T cells and after 24h, precleared lysates of transfected cells were used to immunoprecipitate these Myc-DUBs on anti-Myc beads. Immunoprecipitates were mixed with reaction buffer containing ISG15-VS, and reaction products were analyzed by SDS-PAGE and western blot. Representative of >3 independent, biological replicates.

(b) Same as in (a), except that HA-tagged constructs were used. Representative of >3 independent, biological replicates.

(c) Cell lysates of HEK-293T transfected with the indicated HA-tagged USP41 constructs were separated by SDS-PAGE and analyzed by western blotting. This serves as a control for the experiment presented in Figure 5D.

(d) Protein ISGylation in HEK-293T cells was reconstituted by transfection of the ISG15 machinery (E1/E2/ISG15) and where indicated, HA-tagged constructs were co-transfected. After 24h, lysates of transfected cells were prepared then analyzed by SDS-PAGE and western blot. Immunoblotted antigen is underlined to the left of blots. Representative of 2 independent, biological replicates.

(e) WT, 2CS or L198F versions of HA-USP18A were ectopically expressed in HEK-293T cells, either alone or with 6HF-ISG15. After 24h, precleared lysates of transfected cells were used to immunoprecipitate ISG15 on magnetic anti-Flag beads. Immunoblotted antigen is underlined to the left of blots. Representative of 2 independent, biological replicates.

(f) Cell lysates of HEK-293T transfected with the indicated Myc-tagged USP18 constructs were separated by SDS-PAGE and analyzed by western blotting. This serves as a control for the experiment presented in Figure 5F.

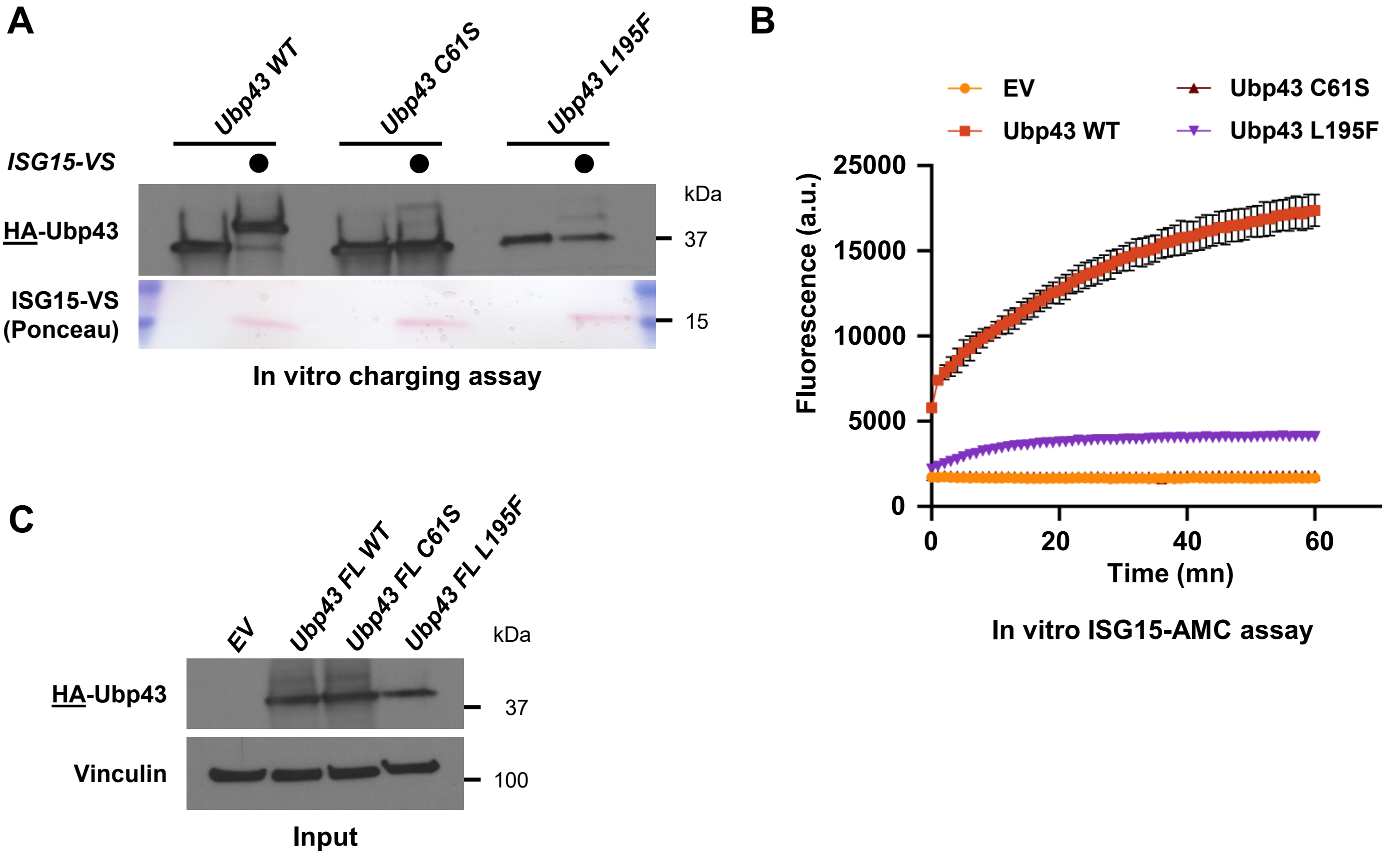

Supplementary Figure 7. Leu195 is important for mUSP18/Ubp43 enzymatic activity.

Supplementary Figure 7. Leu195 is important for mUSP18/Ubp43 enzymatic activity.

(a) The indicated HA-tagged versions of Ubp43 were isolated from HEK-293T cells on anti-HA beads 24h after transfection. HA-Ubp43 immunoprecipitates were mixed with reaction buffer containing ISG15-VS or not, and reaction products were analyzed by SDS-PAGE and western blot. Representative of 2 independent, biological replicates.

(b) The indicated HA-tagged constructs were ectopically expressed in HEK-293T cells and after 24h, precleared lysates of transfected cells were used to immunoprecipitate HA-tagged Ubp43 variants on anti-HA beads. Immunoprecipitates were mixed with reaction buffer containing the fluorogenic substrate ISG15-AMC, and fluorescence increase was monitored as previously described. Representative of >3 independent, biological replicates.

(c) Cell lysates of HEK-293T transfected with the indicated HA-tagged Ubp43 constructs were separated by SDS-PAGE and analyzed by western blotting. This serves as a control for the experiment presented in (b).

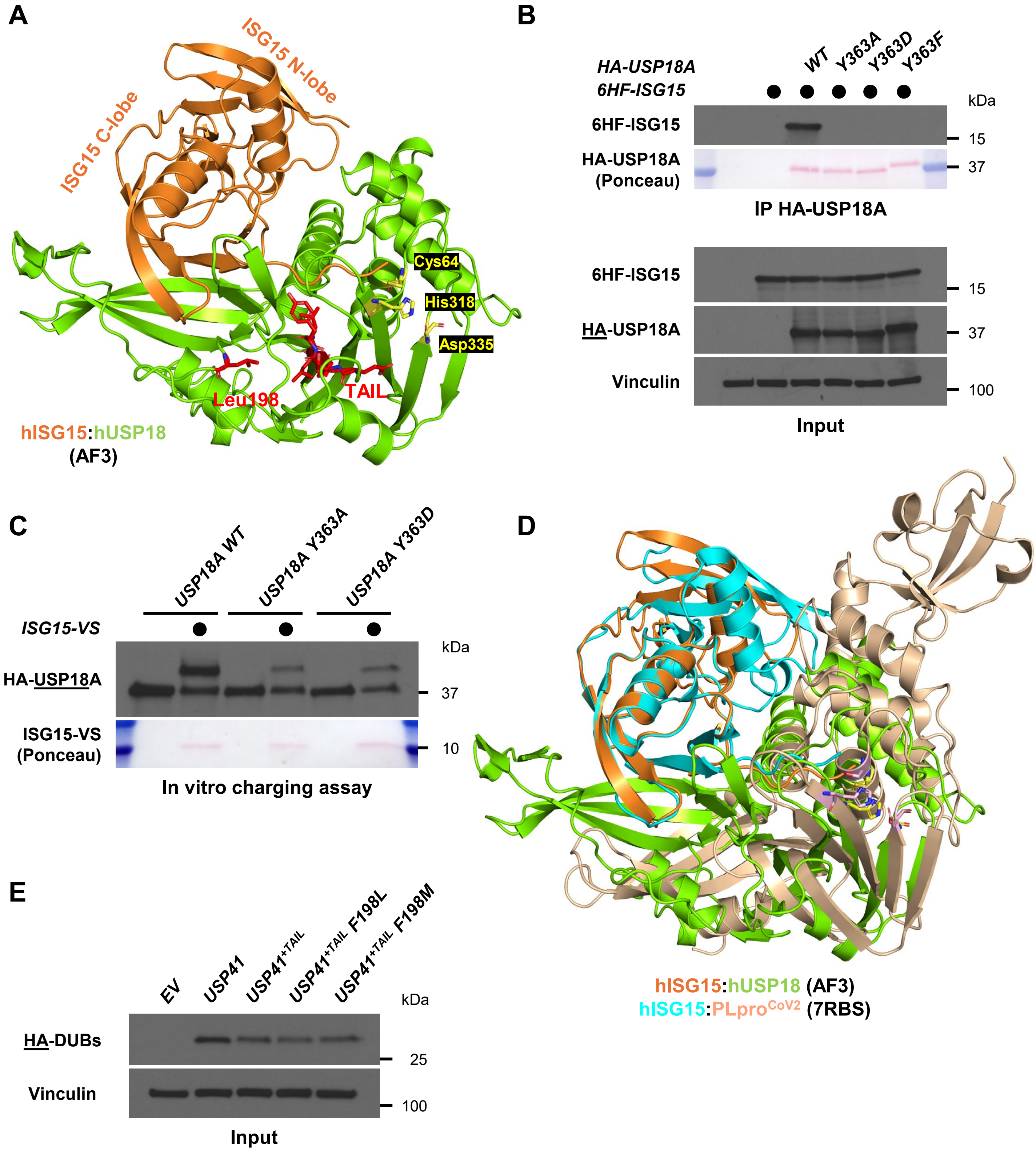

Supplementary Figure 8. Structural analysis of USP18 TAIL motif and Leu198 using AlphaFold 3.

Supplementary Figure 8. Structural analysis of USP18 TAIL motif and Leu198 using AlphaFold 3.

(a) AlphaFold 3 prediction of hUSP18 in complex with hISG15. Leu198 and the TAIL motif are highlighted in red, while the catalytic triad (Cys64, His318, Asn335) is in yellow.

(b) 6HF-ISG15 was ectopically expressed in HEK-293T cells, either alone, with WT or Tyr363 mutant versions of HA-USP18A. At 24h post-transfection, precleared lysates were used to immunoprecipitate HA-USP18 using anti-HA beads. Immunoblotted antigen is underlined to the left of blots. Representative of 2 independent, biological replicates.

(c) The indicated HA-tagged USP18A constructs were purified from HEK-293T cells on anti-HA beads 48h after transfection. HA-USP18 immunoprecipitates were mixed with reaction buffer containing ISG15-VS or not, and reaction products were analyzed by SDS-PAGE and western blot. Representative of >3 independent, biological replicates.

(d) Overlay of the AlphaFold structure of hISG15: hUSP18 with the crystal structure of hISG15:PLpro^{CoV2} (PDB identifier: 7RBS) by aligning the complexes on the C-terminal Ubl domain of ISG15.

(e) Cell lysates of HEK-293T transfected with the indicated HA-tagged USP41 constructs were separated by SDS-PAGE and analyzed by western blotting. This serves a control for the experiment presented in Figure 6F.
