# Supplementary material for: Mechanisms of USP18 specificity toward ISG15 revealed by paralog sequence analysis comparison
Source: J Biol Chem. 2025 May 26;301(7):110288. doi: 10.1016/j.jbc.2025.110288 (PMC12221285; doi:10.1016/j.jbc.2025.110288)
Supplement: Legends Supplementary Tables [file mmc5.docx]

**SUPPLEMENTARY TABLES LEGENDS**

**Supplementary Table 1. IP-MS results of USP18 PTMs identification following 6His-FLAG-ISG15 pull down from HEK-293T cells.**

List of proteins identified by IP-MS following bands cutting and trypsin digestion. Samples 1 and 2 are negative control, while samples 3 and 4 correspond to the upper and lower USP18 isoforms, respectively.

**Supplementary Table 2. USP41 specific peptides identification by MS.**

List of peptides corresponding to both USP18 and USP41, as well as unique USP41 peptides (highlighted in orange).

**Supplementary Table 3. List antibodies, reagents and DNA constructs used and generated for this study.**

List of antibodies and reagents used (first tab) and list of all DNA constructs that were purchased and generated, including their corresponding primers.
