# Supplementary material for: Mechanisms of USP18 specificity toward ISG15 revealed by paralog sequence analysis comparison
Source: J Biol Chem. 2025 May 26;301(7):110288. doi: 10.1016/j.jbc.2025.110288 (PMC12221285; doi:10.1016/j.jbc.2025.110288)
Supplement: Supplementary Document [file mmc6.docx]

**SUPPLEMENTARY INFORMATION- DNA and Protein sequences corresponding to USP18 and USP41**

**USP18 reference sequence (nucleotides) in databases (1119 nt):**
ATGAGCAAGGCGTTTGGGCTCCTGAGGCAAATCTGTCAGTCCATC**CTG**GCTGAGTCCTCGCAGTCCCCGGCAGATCTTGAAGAAAAGAAGGAAGAAGACAGCAAC**ATG**AAGAGAGAGCAGCCCAGAGAGCGTCCCAGGGCCTGGGACTACCCTCATGGCCTGGTTGGTTTACACAACATTGGACAGACCTGCTGCCTTAACTCCTTGATTCAGGTGTTCGTAATGAATGTGGACTTCACCAGGATATTGAAGAGGATCACGGTGCCCAGGGGAGCTGACGAGCAGAGGAGAAGCGTCCCTTTCCAGATGCTTCTGCTGCTGGAGAAGATGCAGGACAGCCGGCAGAAAGCAGTGCGGCCCCTGGAGCTGGCCTACTGCCTGCAGAAGTGCAACGTGCCCTTGTTTGTCCAACATGATGCTGCCCAACTGTACCTCAAACTCTGGAACCTGATTAAGGACCAGATCACTGATGTGCACTTGGTGGAGAGACTGCAGGCCCTGTATACGATCCGGGTGAAGGACTCCTTGATTTGCGTTGACTGTGCCATGGAGAGTAGCAGAAACAGCAGCATGCTCACCCTCCCACTTTCTCTTTTTGATGTGGACTCAAAGCCCCTGAAGACACTGGAGGACGCCCTGCACTGCTTCTTCCAGCCCAGGGAGTTATCAAGCAAAAGCAAGTGCTTCTGTGAGAACTGTGGGAAGAAGACCCGTGGGAAACAGGTCTTGAAGCTGACCCATTTGCCCCAGACCCTGACAATCCACCTCATGCGATTCTCCATCAGGAATTCACAGACGAGAAAGATCTGCCACTCCCTGTACTTCCCCCAGAGCTTGGATTTCAGCCAGATCCTTCCAATGAAGCGAGAGTCTTGTGATGCTGAGGAGCAGTCTGGAGGGCAGTATGAGCTTTTTGCTGTGATTGCGCACGTGGGAATGGCAGACTCCGGTCATTACTGTGTCTACATCCGGAATGCTGTGGATGGAAAATGGTTCTGCTTCAATGACTCCAATATTTGCTTGGTGTCCTGGGAAGACATCCAGTGTACCTACGGAAATCCTAACTACCACTGGCAGGAAACTGCATATCTTCTGGTTTACATGAAGATGGAGTGCTAA

Transcription initiation sites to generate USP18A (CTG) or USP18B (ATG)

**USP18 reference sequence (amino acids) in databases (1-372)**

MSKAFGLLRQICQSILAESSQSPADLEEKKEEDSNMKREQPRERPRAWDYPHGLVGLHNIGQTCCLNSLIQVFVMNVDFTRILKRITVPRGADEQRRSVPFQMLLLLEKMQDSRQKAVRPLELAYCLQKCNVPLFVQHDAAQLYLKLWNLIKDQITDVHLVERLQALYTIRVKDSLICVDCAMESSRNSSMLTLPLSLFDVDSKPLKTLEDALHCFFQPRELSSKSKCFCENCGKKTRGKQVLKLTHLPQTLTIHLMRFSIRNSQTRKICHSLYFPQSLDFSQILPMKRESCDAEEQSGGQYELFAVIAHVGMADSGHYCVYIRNAVDGKWFCFNDSNICLVSWEDIQCTYGNPNYHWQETAYLLVYMKMEC

Catalytic triad

**USP18A nucleotide sequence (1074 nt):**
**CTG**GCTGAGTCCTCGCAGTCCCCGGCAGATCTTGAAGAAAAGAAGGAAGAAGACAGCAAC**ATG**AAGAGAGAGCAGCCCAGAGAGCGTCCCAGGGCCTGGGACTACCCTCATGGCCTGGTTGGTTTACACAACATTGGACAGACCTGCTGCCTTAACTCCTTGATTCAGGTGTTCGTAATGAATGTGGACTTCACCAGGATATTGAAGAGGATCACGGTGCCCAGGGGAGCTGACGAGCAGAGGAGAAGCGTCCCTTTCCAGATGCTTCTGCTGCTGGAGAAGATGCAGGACAGCCGGCAGAAAGCAGTGCGGCCCCTGGAGCTGGCCTACTGCCTGCAGAAGTGCAACGTGCCCTTGTTTGTCCAACATGATGCTGCCCAACTGTACCTCAAACTCTGGAACCTGATTAAGGACCAGATCACTGATGTGCACTTGGTGGAGAGACTGCAGGCCCTGTATACGATCCGGGTGAAGGACTCCTTGATTTGCGTTGACTGTGCCATGGAGAGTAGCAGAAACAGCAGCATGCTCACCCTCCCACTTTCTCTTTTTGATGTGGACTCAAAGCCCCTGAAGACACTGGAGGACGCCCTGCACTGCTTCTTCCAGCCCAGGGAGTTATCAAGCAAAAGCAAGTGCTTCTGTGAGAACTGTGGGAAGAAGACCCGTGGGAAACAGGTCTTGAAGCTGACCCATTTGCCCCAGACCCTGACAATCCACCTCATGCGATTCTCCATCAGGAATTCACAGACGAGAAAGATCTGCCACTCCCTGTACTTCCCCCAGAGCTTGGATTTCAGCCAGATCCTTCCAATGAAGCGAGAGTCTTGTGATGCTGAGGAGCAGTCTGGAGGGCAGTATGAGCTTTTTGCTGTGATTGCGCACGTGGGAATGGCAGACTCCGGTCATTACTGTGTCTACATCCGGAATGCTGTGGATGGAAAATGGTTCTGCTTCAATGACTCCAATATTTGCTTGGTGTCCTGGGAAGACATCCAGTGTACCTACGGAAATCCTAACTACCACTGGCAGGAAACTGCATATCTTCTGGTTTACATGAAGATGGAGTGCTAA

**USP18A amino acid sequence (16-372)**

MAESSQSPADLEEKKEEDSNMKREQPRERPRAWDYPHGLVGLHNIGQTCCLNSLIQVFVMNVDFTRILKRITVPRGADEQRRSVPFQMLLLLEKMQDSRQKAVRPLELAYCLQKCNVPLFVQHDAAQLYLKLWNLIKDQITDVHLVERLQALYTIRVKDSLICVDCAMESSRNSSMLTLPLSLFDVDSKPLKTLEDALHCFFQPRELSSKSKCFCENCGKKTRGKQVLKLTHLPQTLTIHLMRFSIRNSQTRKICHSLYFPQSLDFSQILPMKRESCDAEEQSGGQYELFAVIAHVGMADSGHYCVYIRNAVDGKWFCFNDSNICLVSWEDIQCTYGNPNYHWQETAYLLVYMKMEC

Catalytic triad

**USP18B nucleotide sequence (1014 nt):**
**ATG**AAGAGAGAGCAGCCCAGAGAGCGTCCCAGGGCCTGGGACTACCCTCATGGCCTGGTTGGTTTACACAACATTGGACAGACCTGCTGCCTTAACTCCTTGATTCAGGTGTTCGTAATGAATGTGGACTTCACCAGGATATTGAAGAGGATCACGGTGCCCAGGGGAGCTGACGAGCAGAGGAGAAGCGTCCCTTTCCAGATGCTTCTGCTGCTGGAGAAGATGCAGGACAGCCGGCAGAAAGCAGTGCGGCCCCTGGAGCTGGCCTACTGCCTGCAGAAGTGCAACGTGCCCTTGTTTGTCCAACATGATGCTGCCCAACTGTACCTCAAACTCTGGAACCTGATTAAGGACCAGATCACTGATGTGCACTTGGTGGAGAGACTGCAGGCCCTGTATACGATCCGGGTGAAGGACTCCTTGATTTGCGTTGACTGTGCCATGGAGAGTAGCAGAAACAGCAGCATGCTCACCCTCCCACTTTCTCTTTTTGATGTGGACTCAAAGCCCCTGAAGACACTGGAGGACGCCCTGCACTGCTTCTTCCAGCCCAGGGAGTTATCAAGCAAAAGCAAGTGCTTCTGTGAGAACTGTGGGAAGAAGACCCGTGGGAAACAGGTCTTGAAGCTGACCCATTTGCCCCAGACCCTGACAATCCACCTCATGCGATTCTCCATCAGGAATTCACAGACGAGAAAGATCTGCCACTCCCTGTACTTCCCCCAGAGCTTGGATTTCAGCCAGATCCTTCCAATGAAGCGAGAGTCTTGTGATGCTGAGGAGCAGTCTGGAGGGCAGTATGAGCTTTTTGCTGTGATTGCGCACGTGGGAATGGCAGACTCCGGTCATTACTGTGTCTACATCCGGAATGCTGTGGATGGAAAATGGTTCTGCTTCAATGACTCCAATATTTGCTTGGTGTCCTGGGAAGACATCCAGTGTACCTACGGAAATCCTAACTACCACTGGCAGGAAACTGCATATCTTCTGGTTTACATGAAGATGGAGTGCTAA

**USP18B amino acid sequence (36-372)**

MKREQPRERPRAWDYPHGLVGLHNIGQTCCLNSLIQVFVMNVDFTRILKRITVPRGADEQRRSVPFQMLLLLEKMQDSRQKAVRPLELAYCLQKCNVPLFVQHDAAQLYLKLWNLIKDQITDVHLVERLQALYTIRVKDSLICVDCAMESSRNSSMLTLPLSLFDVDSKPLKTLEDALHCFFQPRELSSKSKCFCENCGKKTRGKQVLKLTHLPQTLTIHLMRFSIRNSQTRKICHSLYFPQSLDFSQILPMKRESCDAEEQSGGQYELFAVIAHVGMADSGHYCVYIRNAVDGKWFCFNDSNICLVSWEDIQCTYGNPNYHWQETAYLLVYMKMEC

Catalytic triad

**USP41 nucleotide sequence (1077 nt):**

ATGGACGGCGTCTTGTTCAGGGCTCATCAGTGTCAGTACGTGCATCCTTGTGTACATGTGTATGTCACTGTGGGCCTCATGGATCCCCTGTGCGAAAGAAAAGAAAAAGCAAGCAAGCAAGAGAGAGAAAACCCCTTGGCCCACCTAGCAGCATGGGGCCTGGTTGGTTTACACAACATTGGACAGACCTGCTGCCTTAACTCCTTGATTCAGGTGTTTGTAATGAATGTGGACTTCGCCAGGATATTGAAGAGGATCACGGTGCCCAGGGGAGCTGACGAGCAGAGGAGAAGCGTCCCTTTCCAGATGCTTCTGCTGCTGGAGAAGATGCAGGACAGCCGGCAGAAAGCAGTGTGGCCCCTGGAGCTGGCCTACTGCCTGCAGAAGTACAACGTGCCCCTGTTTGTCCAACATGATGCTGCCCAACTGTACCTCAAACTCTGGAACCTGATTAAGGACCAGATCGCTGATGTGCACTTGGTGGAGAGACTGCAGGCCCTGTATATGATCCGGATGAAGGACTCCTTGATTTGCCTTGACTGTGCCATGGAGAGTAGCAGAAACAGCAGCATGCTCACCCTCCGACTTTCTTTTTTTGATGTGGACTCAAAGCCCCTGAAGACACTGGAGGACGCCCTGCACTGCTTCTTCCAGCCCAGGGAGTTATCAAGCAAAAGCAAATGCTTCTGTGAGAACTGTGGGAAGAAGACCCGTGGGAAACAGGTCTTGAAGCTGACCCATTTGCCCCAGACCCTGACAATCCACCTCATGCGATTCTCCATCAGGAATTCACAGACGAGAAAGATCTGCCACTCCCTGTACTTCCCCCAGAGCTTGGATTTCAGCCAGATCCTTCCAATGAAGCGAGAGTCTTGTGATGCTGAGGAGCAGTCTGGAGGGCAGTATGAGCTTTTTGCTGTGATTGCGCACGTGGGAATGGCAGACTCCGGTCATTACTGTGTCTACATCCGGAATGCTGTGGATGGAAAATGGTTCTGCTTCAATGACTCCAATATTTGCTTGGTGTCCTGGGAAGACATCCAGTGTACCTACGGAAATCCTAACTACCACTGGTAA

**USP41 amino acid sequence (1-358)**

MDGVLFRAHQCQYVHPCVHVYVTVGLMDPLCERKEKASKQERENPLAHLAAWGLVGLHNIGQTCCLNSLIQVFVMNVDFARILKRITVPRGADEQRRSVPFQMLLLLEKMQDSRQKAVWPLELAYCLQKYNVPLFVQHDAAQLYLKLWNLIKDQIADVHLVERLQALYMIRMKDSLICLDCAMESSRNSSMLTLRLSFFDVDSKPLKTLEDALHCFFQPRELSSKSKCFCENCGKKTRGKQVLKLTHLPQTLTIHLMRFSIRNSQTRKICHSLYFPQSLDFSQILPMKRESCDAEEQSGGQYELFAVIAHVGMADSGHYCVYIRNAVDGKWFCFNDSNICLVSWEDIQCTYGNPNYHW

Predicted catalytic triad
